# Highly Self‐Adhesive and Biodegradable Silk Bioelectronics for All‐In‐One Imperceptible Long‐Term Electrophysiological Biosignals Monitoring

**DOI:** 10.1002/advs.202405988

**Published:** 2025-01-10

**Authors:** Seyed Sajjad Mirbakht, Ata Golparvar, Muhammad Umar, Burcu Arman Kuzubasoglu, Farid Sayar Irani, Murat Kaya Yapici

**Affiliations:** ^1^ Faculty of Engineering and Natural Sciences Sabanci University Istanbul 34956 Türkiye; ^2^ Sabanci University Micro/Nano Devices and Systems Lab (SU‐MEMS) Sabanci University Istanbul 34956 Türkiye; ^3^ ICLab École Polytechnique Fédérale de Lausanne (EPFL) Neuchâtel 2002 Switzerland; ^4^ Sabanci University SUNUM Nanotechnology Research Center Istanbul 34956 Türkiye; ^5^ Department of Electrical Engineering University of Washington Seattle WA 98195 USA

**Keywords:** biopotential monitoring, flexible electronics, functional biomaterials, stretchable electronics, wearable silk

## Abstract

Skin‐like bioelectronics offer a transformative technological frontier, catering to continuous and real‐time yet highly imperceptible and socially discreet digital healthcare. The key technological breakthrough enabling these innovations stems from advancements in novel material synthesis, with unparalleled possibilities such as conformability, miniature footprint, and elasticity. However, existing solutions still lack desirable properties like self‐adhesivity, breathability, biodegradability, transparency, and fail to offer a streamlined and scalable fabrication process. By addressing these challenges, inkjet‐patterned protein‐based skin‐like silk bioelectronics (Silk‐BioE) are presented, that integrate all the desirable material features that have been individually present in existing devices but never combined into a single embodiment. The all‐in‐one solution possesses excellent self‐adhesiveness (300 N m^−1^) without synthetic adhesives, high breathability (1263 g h^−1^ m^−2^) as well as swift biodegradability in soil within a mere 2 days. In addition, with an elastic modulus of ≈5 kPa and a stretchability surpassing 600%, the soft electronics seamlessly replicate the mechanics of epidermis and form a conformal skin/electrode interface even on hairy regions of the body under severe perspiration. Therefore, coupled with a flexible readout circuitry, Silk‐BioE can non‐invasively monitor biosignals (i.e., ECG, EEG, EOG) in real‐time for up to 12 h with benchmarking results against Ag/AgCl electrodes.

## Graphical abstract

Skin‐like bioelectronics offer continuous and imperceptible digital healthcare, yet existing solutions lack key material properties. The inkjet‐patterned silk (Silk‐BioE), with high self‐adhesiveness (300 N m^−1^), breathability (1263 g h^−1^ m^−2^), biodegradability, and 600% stretchability monitors biosignals (i.e., ECG, EEG, EOG) with a flexible readout circuitry in real‐time, even on hairy regions of the body under severe perspiration.

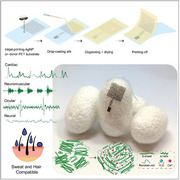

## 1 Introduction

Stretchable, skin‐like electronics offer a versatile approach to non‐invasive skin interfacing, enabling the acquisition of crucial physiological health data or the delivery of therapeutic stimulation and medication.^[^ ^1^ ^]^ In contrast to conventional rigid technologies, these platforms possess mechanical characteristics akin to the epidermis,^[^ ^2^ ^]^ and this seamless conformal interface is instrumental in high‐fidelity biosignal acquisition while minimizing susceptibility to motion artifacts.^[^ ^3^, ^4^, ^5^ ^]^ Within healthcare, their applications span a wide spectrum, encompassing the evaluation of skin thermal variations,^[^ ^6^ ^]^ innovative wound dressing techniques,^[^ ^7^ ^]^ monitoring skin hydration levels,^[^ ^8^ ^]^ assessing sleep quality,^[^ ^9^ ^]^ detecting biomarkers directly on the skin surface,^[^ ^10^ ^]^ and multifunctional detection with electromagnetic shielding properties.^[^ ^11^, ^12^ ^]^ In soft robotics, these devices have played a pivotal role in providing sensory feedback for prosthetic hands^[^ ^13^ ^]^ and enabling electro‐tactile stimulation for interactive human‐robot interfaces.^[^ ^14^ ^]^ Their utility extends further into self‐powered electronic devices through energy harvesting methods.^[^ ^15^, ^16^ ^]^

The fabrication of skin‐like bioelectronics, particularly those utilizing photolithography‐based cleanroom procedures, has been instrumental in propelling the field forward due to its ability to achieve high patterning resolution and quality. However, the potential for exploring alternative methods is hinted at by the intricate and resource‐intensive nature of these processes, especially when applied to products designed for single and disposable use.^[^ ^17^, ^18^ ^]^ This is because cleanroom processes demand a controlled environment, the establishment and maintenance of which can incur substantial costs and energy. The inherent complexity of these procedures may result in increased costs and challenges in maintaining precise control, potentially complicating the fabrication process and inflating the final product's cost, thereby limiting their accessibility for broader use.^[^ ^19^, ^20^ ^]^ Given the unique opportunities presented by alternative, cleanroom‐free additive fabrication techniques, the transition of skin‐like electronics from academic research prototypes to clinical‐grade scalable technologies is feasible. This could potentially benefit a significant segment of the population as well as maintaining an environmentally friendly production cycle.

Indeed, concerns have also been raised regarding the environmental impact of chemically synthesized plastics and polymers.^[^ ^21^, ^22^, ^23^, ^24^, ^25^, ^26^ ^]^ These materials often endure prolonged natural degradation when disposed of after a single use. Compounding this issue is the escalating volume of disposable electronics, contributing substantially to the burgeoning problem of electronic waste (e‐waste), which is projected to reach a staggering 74 million tons by 2030.^[^ ^27^ ^]^

Another challenge shared between all skin‐interfaced wearables persists in the limited biocompatibility of skin‐like bioelectronics, posing potential discomfort and dermatological irritation with extended use. Such discomfort may stem from using non‐organic or potentially hazardous materials and disrupting the skin's natural trans‐epidermal water loss and air permeation.^[^ ^28^ ^]^ Furthermore, the continuous exposure of skin electronics to the natural deformation of the skin, sweat, and oils poses a significant challenge. Weak bonding between these devices and the skin often results in detachment, leading to performance issues such as data loss during signal recording and interruptions in continuous monitoring. This problem is particularly pronounced for skin electronics relying on low Van der Waals forces for adhesion, owing to their inherently lower adhesive energy.^[^ ^29^ ^]^

Addressing these issues is imperative to ensure that skin‐like bioelectronics sustains functionality and comfort for continuous long‐term medical data collection and vital sign tracking applications. Biopolymers, including silk,^[^ ^30^ ^]^ cellulose,^[^ ^31^ ^]^ chitin,^[^ ^32^ ^]^ and lignin,^[^ ^33^ ^]^ are gaining recognition as robust biomaterials for the development of bioelectronics, owing to their inherent biocompatibility^[^ ^34^, ^35^, ^36^ ^]^ and widespread availability.^[^ ^37^, ^38^ ^]^ Silk is a prime candidate for diverse medical applications due to its distinct natural structure, tunable mechanical properties,^[^ ^39^ ^]^ self‐healing properties,^[^ ^40^, ^41^, ^42^, ^43^ ^]^ and degradation characteristics.^[^ ^44^ ^]^ Various formats of silk, such as sponges,^[^ ^45^ ^]^ hydrogels,^[^ ^46^ ^]^ patches,^[^ ^47^ ^]^ and fibers,^[^ ^48^ ^]^ have been explored for applications in drug delivery,^[^ ^49^ ^]^ wound healing,^[^ ^50^ ^]^ and biosignal acquisition.^[^ ^51^ ^]^

Recent advancements have precisely leveraged calcium ions (Ca^2+)^ to tailor silk properties, such as modulus, adhesiveness, and degradation rate, enhancing its suitability for skin‐like electronics.^[^ ^35^, ^39^, ^52^ ^]^ These properties render silk an ideal substrate for such applications when properly tuned. For instance, the self‐adhesive nature of silk‐based skin‐like bioelectronics plays a pivotal role in continuous physiological signal recording.^[^ ^53^ ^]^ However, while robust adhesion is essential for accurate data capture, it can pose challenges, especially for individuals with delicate skin, such as the elderly and newborns. Their thinner skin layers and higher transepidermal water loss make their skin more susceptible to irritation and damage.^[^ ^54^, ^55^, ^56^ ^]^ Balancing water solubility and adhesiveness through meticulous material property adjustments is key. This ensures adequate adherence of the electronics to the skin while enabling easy removal through simple washing, thereby mitigating potential skin damage. Achieving this balance holds promise in preserving both the efficacy of data capture and the skin health of vulnerable individuals.

Therefore, this work introduces stretchable, skin‐like silk bioelectronics (Silk‐BioE) for wireless and continuous human physiological sensing. Through precise tuning of the protein structure of the silk, process engineering, deposition, patterning optimization, and electronic circuitry design, we have developed a comprehensive electrophysiological system while wirelessly transmitting data. Our device integrates Ca^2+^‐modified silk films with fine silver nanoparticles (AgNP) serpentine microstructures additively patterned using inkjet printing, achieving the desired modulus, stretchability, adhesion, degradability, and transparency for skin‐like epidermal bioelectronics. The flexible and wireless integrated electronic system interfaces seamlessly with the ultrasoft silk electronics, encompassing necessary amplification, processing, and wireless data transmission. This integration eliminates the need for extra wiring and bulky hardware, providing discreet access to vital human health information, including cardiovascular performance, brain activity, eyeball orientation for computer‐machine interfaces, and muscle fatigue detection.

## 2 Results

### 2.1 Skin‐Like Silk Bioelectronics (Silk‐BioE)

**Figure 1:**
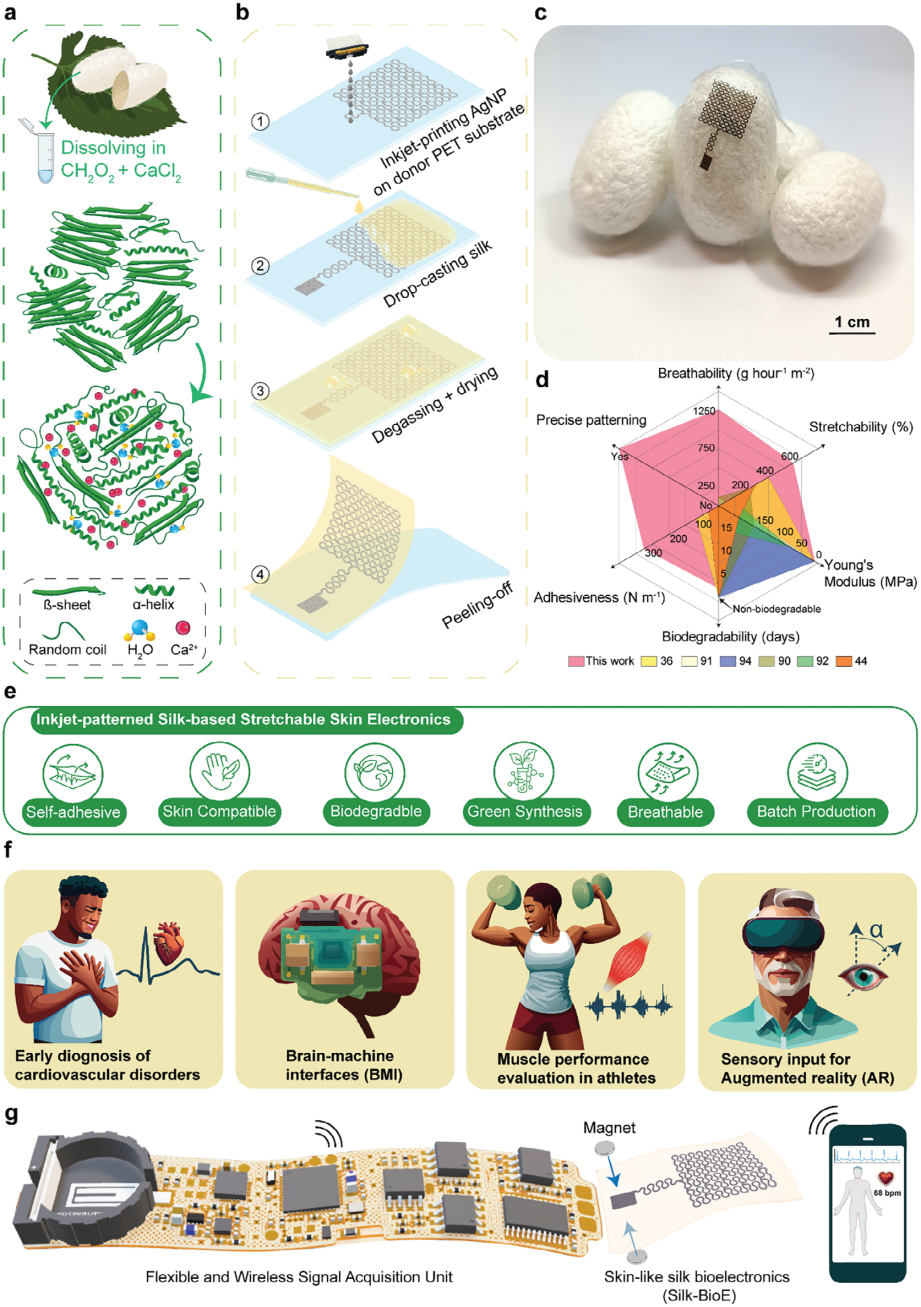
Schematic illustration of the synthesis, fabrication, and application of the introduced skin‐like silk bioelectronics (Silk‐BioE). a) Graphical representation of the effect of Ca^2+^ on the protein structure transition from crystalline to amorphous. b) Silk‐BioE patterning: (1) inkjet‐printing of AgNP on donor PET substrate, (2) drop‐casting silk solution on the printed AgNP mesh, (3) degassing and drying of the silk solution, and (4) transferring the AgNP pattern to silk by peeling the silk from PET donor substrate. c) Photographic depiction of the Silk‐BioE positioned on the original *Bombyx mori* silk cocoons. d) Comparative analysis of Silk‐BioE and existing silk‐based skin electronics for physiological monitoring. Details are given in Table 1. e) Schematic representation of key features of Silk‐BioE. f) Potential applications of the unit from healthcare to human‐computer interaction (HCI) to fitness and wellness to emerging novel applications in virtual reality. g) A prototype of the Silk‐BioE connected with a flexible wireless electronic circuitry.

**Figure** **1**a illustrates the process of silk proteins undergoing molecular self‐assembly by the influence of calcium ions (Ca^2+^). The synthesis begins with dissolving *Bombyx mori* silk cocoons in a solution composed of calcium chloride (CaCl_2_) and formic acid (HCOOH),^[^ ^39^ ^]^ following the degumming procedure as detailed in the experimental section (Figure S1, Supporting Information). The CaCl_2_ introduces Ca^2+^, which enhances the stretchability of the silk films by diminishing the crystalline nature of the initial rigid silk structure.^[^ ^57^ ^]^ This transformation signifies a shift from a dominant β‐sheet protein structure to a heightened presence of random coils and α‐helices (verified by Fourier‐transform infrared spectroscopy, FTIR, discussed in the next section).^[^ ^35^, ^39^ ^]^

Figure 1b illustrates transferring inkjet‐printed patterns onto a silk substrate. The process initiates with 1) inkjet printing a serpentine network made of AgNP onto donor polyethylene terephthalate (PET) substrates, followed by 2) drop‐casting the silk solution on the printed AgNP patterns. Subsequently, 3) a single‐step low‐temperature heating process at 75 °C is employed to temporarily stiffen the silk films, facilitating the 4) efficient transfer of the printed patterns from the donor substrates to the silk films (Figure 1c). To evaluate the fidelity of the printed AgNP structure prior to and following transfer printing, the resistance of a serpentine resistive design was measured on the donor PET substrate and on the silk substrate after transfer. The results (Figure S2, Supporting Information) show a slight variation of ≈1.25% in the resistance of the AgNP pattern before (279.4 Ω) and after (282.9 Ω) the transfer, indicating that minimal damage was sustained by the printed AgNP patterns during the transfer process. The serpentine network, with a width of 200 µm, plays a crucial role in enhancing the mechanical and electrical properties of the patch. This is associated with the curved nature of the design, which alleviates induced mechanical stress, ensuring the stable collection of biopotential signals under various skin deformations.^[^ ^58^ ^]^ Figure 1d provides a quantitative and qualitative comparison of Silk‐BioE with previously reported silk‐based skin electronics used for physiological monitoring. For biosignal recording, the Silk‐BioE connects with a flexible and compact (15 × 75 mm) wireless data acquisition system (Figure 1g), facilitating multimodal monitoring of various non‐invasive human electrophysiological biosignals that serve for broad applications in wearable technologies, spanning from digital healthcare, wellness, and fitness to human‐computer interaction, and to provide new sensory inputs to advance augmented reality systems (Figure 1f).

### 2.2 Development and Characterization of Pre‐Printed Pristine Silk Films

**Figure 2:**
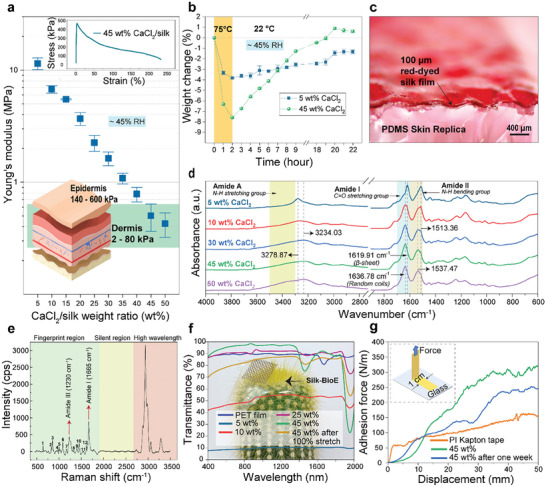
Characterization of silk as a soft, self‐adhesive, biodegradable, transparent, and stretchable substrate for skin‐like bioelectronics. a) The Young's modulus of the silk substrate corresponds to different CaCl_2_ values at a relative humidity of ≈45% (*n* = 5), where the inset shows the tensile stress‐strain curve of 45 wt.% CaCl_2_/silk weight ratio. b) The strength of losing and capturing the ambient water molecules of 5 and 45 wt.% silk substrates (*n* = 3). c) The interface of red‐dyed 100 µm silk substrate with PDMS skin replica. d) FTIR spectra of different CaCl_2_/silk ratios illustrate the effect of Ca^2+^ ions on forming beta‐sheets and random coils. e) Raman spectra of the 45 wt.% CaCl_2_/silk substrate. f) Transmittance analysis of the silk substrates with different CaCl_2_/silk weight ratios. g) Adhesion force of silk substrates and commercial Kapton tape with respect to glass.

The mechanical properties of the silk films were fine‐tuned through the controlled introduction of CaCl_2_ to the silk fibroin fibers, which enabled their transformation into ultra‐soft and imperceptible substrates for epidermal bioelectronics. The Young's modulus of the silk films exhibited a significant reduction, decreasing from 11.4 MPa to 427.1 kPa, as the CaCl_2_ to silk weight ratio increased from 5 to 50 wt.% at a relative humidity of ≈45% RH (**Figure** **2**a). Correspondingly, the stretchability of the silk films increased substantially, expanding from a mere 6% to an impressive >250% (Figure S3, Supporting Information). However, the silk films loaded with 50 wt.% CaCl_2_, despite their lower modulus of elasticity, were found to be mechanically unstable as a stand‐alone solid substrate. Under ≈45% humidity, they exhibited properties akin to a gel, rendering them impractical as a structural support for attachment onto the skin. In contrast, silk films loaded with 45 wt.% CaCl_2_ maintained superior mechanical characteristics, featuring Young's modulus of 501.1 kPa and stretchability of >230% (Figure 2a inset). These properties closely matched the mechanical attributes of human skin, specifically the epidermis (which typically has a modulus ranging from 140 to 600 kPa^[^ ^59^, ^60^ ^]^). Consequently, the subsequent experiments in this study were conducted using silk films loaded with 45 wt.% CaCl_2_.

The influence of ambient humidity on the silk films is of notable significance, given that silk can absorb more water molecules as the Ca^2+^ ion content increases. This is due to the high coordination number of calcium ions (Ca^2+^), which typically bind with 6–8 water molecules in their primary hydration shell. This extensive coordination capacity allows Ca^2+^ to attract and retain a substantial number of water molecules. Additionally, the relatively low hydration energy of Ca^2+^ makes the binding of multiple water molecules energetically favorable. Consequently, the presence of Ca^2+^ ions increases the overall water content in silk films.^[^ ^61^, ^62^ ^]^ For instance, the modulus of the 45wt.% CaCl_2_/silk films decreased from 501.1 to 5.1 kPa, and the stretchability increased from >230% to >600% as the humidity was varied from ≈45% to ≈60% RH in a custom‐designed humidity chamber into which the silk films were placed (Figure S4, Supporting Information). This phenomenon represents an additional adjustable parameter for fine‐tuning the elasticity of Ca^2+^‐modified silk films.

Moreover, silk films show revertability to their original form by reabsorbing water content after experiencing water loss. In the case of 45 wt.% CaCl_2_ loaded silk, a weight loss of 7.6% measured by a high precision (0.1 mg) scale was observed following a 2‐h exposure to 75 °C in a convection oven. Remarkably, this weight loss was fully recovered after 19 h, and the material exceeded its initial weight by ≈0.7% after 22 h (Figure 2b). This indicates that the reported silk films can recover after undergoing high temperatures and regain their elasticity. In contrast, silk with 5 wt.% CaCl_2_ exhibited a weight loss of only 3.8% and did not return to its original weight even after 22 h. These weight fluctuations are attributed to variations in water content, due to presence of Ca^2+^, which, in turn, have a discernible impact on the modulus of the silk films.

Figure 2c illustrates the elasticity and softness of a 45 wt.% CaCl_2_‐loaded red‐dyed silk film on a Polydimethylsiloxane (PDMS) skin replica. The interface between the silk and the skin replica demonstrates the tight conformality of the produced silk films in mimicking the surface topography of the epidermis. Indeed, it is essential to have a conformal electrode/skin interface to enhance the stability of the signal recording and reduce motion artifacts, which are the typical limiting factors of the recorded signal signal‐to‐noise ratio (SNR).^[^ ^63^ ^]^ The surface roughness of the PDMS skin replica is represented with scanning electron microscope (SEM) images with an estimated pore size of ≈180 µm. (Figure S5, Supporting Information).

Figure 2d presents the characterization of the molecular structures of Ca^2+^‐modified silk substrates, with varying CaCl_2_/silk ratios, using FTIR spectroscopy. In the amide I region (1600–1700 cm^−1^, C≐O stretching group), bands at 1619.91 and 1636.78 cm^−1^ are assigned to the β‐sheet and random coil structures, respectively. With the gradual increase in CaCl_2_ content from 5 to 50 wt.%, a noticeable transformation was observed: the β‐sheet structures gradually transitioned into random coils. This transformation signifies the shift from a crystalline form to an amorphous one brought about by the interaction of Ca^2+^ ions.^[^ ^64^ ^]^ Furthermore, the amide II peak (1500–1600 cm^−1^ region, associated with N–H bending groups) displayed a blueshift from 1513 to 1537 cm^−1^ as the CaCl_2_ ratio increased from 5 to 50 wt.%. These amide I and II bands are linked to the backbone conformation of the polymer chain and are indicative of the secondary protein structure.^[^ ^65^ ^]^ Additionally, the amide A peak (3300–3500 cm^−1^ region, corresponding to N‐H stretching groups^[^ ^66^ ^]^) experienced a redshift, shifting from 3278 cm^−1^ in 5 wt.% CaCl_2_ silk to 3234 cm^−1^ in 50 wt.% silk. This shift was attributed to the transition from hydrogen bonding to forming Ca‐N bonds through chelate reactions.^[^ ^53^, ^67^ ^]^ To further validate this transition from crystalline to amorphous structural alterations, X‐ray diffraction (XRD) data (Figure S6, Supporting Information) were examined. These results revealed a broad peak at 26.54° for the 45 wt.% compositions, while two peaks were observed at 9.008° and 20.23° in the 2θ scattering angle range for the 5 wt.% CaCl_2_/silk compositions, verifying the transition from crystalline to amorphous structure of the silk protein structures.^[^ ^68^, ^69^ ^]^ Figure 2e presents the Raman spectra of the silk substrate, highlighting the amide I and amide III Raman shifts at 1665 and 1230 cm^−1^, respectively. These shifts offer valuable insights into the secondary structure of silk. Furthermore, the Raman spectra also allow for the extraction of information regarding the amino acid composition of the silk, as detailed in Table S1 (Supporting Information).^[^ ^70^ ^]^

To investigate the biodegradability of the silk substrates, the films were modified with 45 and 5 wt.% CaCl_2_, both with a thickness of 100 µm, were subjected to soil burial and monitored after 2 days. We show that the 45 wt.% silk films achieved complete decomposition in the soil within only 2 days (Figure S7 and Movie S1, Supporting Information). Indeed, this rapid degradation aligns with the FTIR analysis. The prevalence of β‐sheet structures in the 5 wt.% CaCl_2_ silk films extended the degradation timeline compared to the 45 wt.% CaCl_2_ silk films, where random coils dominated the structural composition.^[^ ^71^ ^]^ The remarkable biodegradability exhibited by silk establishes it as an alternative to the commonly used non‐biodegradable synthetic polymers for applications in skin‐like bioelectronics.^[^ ^21^, ^24^, ^72^, ^73^ ^]^

Any epidermal bioelectronic device may be exposed to high humidity or perspiration and must maintain its structural integrity to ensure optimal performance for long‐term monitoring applications (beyond controlled lab conditions). Therefore, water‐solubility for the silk films was investigated. A 100 µm‐thick, red‐dyed silk film containing 45 wt.% CaCl_2_ was subjected to stirring in deionized (DI) water for 5 h. The silk film did not dissolve during this period (Figure S8, Supporting Information).

Furthermore, the wettability of the silk films was investigated by measuring the contact angle for silk films with varying CaCl_2_/silk ratios. We observed that higher concentrations of CaCl_2_ resulted in greater hydrophilicity of the silk films (Figure S9, Supporting Information). This enhanced hydrophilicity was attributed to the higher water content in the 50 wt.% CaCl_2_/silk films than the 5 wt.% counterparts.^[^ ^74^ ^]^

Another vital biomaterial attribute is transparency which allows epidermal bioelectronics to blend in naturally with the skin tone and reduce their noticeable appearance when worn, where the acceptance and comfort of the user depend on this integration of aesthetics. Figure 2f analyzes the transparency of 100 µm silk films with varying CaCl_2_ ratios. The transmission of these silk films decreased significantly from an initial level of over 95% to 8% as the CaCl_2_/silk ratio was reduced from 45 to 5 wt.%. This decline in transmission for the silk films with lower CaCl_2_ loading was attributed to light scattering originating from larger crosslinking sites predominantly consisting of β‐sheet structures.^[^ ^75^ ^]^ Furthermore, the developed silk films exhibited higher transparency than widely‐used PET films in wearable devices by ≈7%.^[^ ^76^, ^77^, ^78^ ^]^ Moreover, in the event of 100% stretching of the 45 wt.% CaCl_2_/silk film, the decrease in transparency of 95 to 78% was negligible. Additionally, the transparency of 100 µm‐thick silk films with CaCl_2_ concentrations of 5, 10, 25, and 45 wt.% was optically characterized (Figure S10, Supporting Information). The results indicate that as the CaCl_2_ concentration increases, the transparency of the films also increases, with the 5 wt.% CaCl_2_/silk film being the most opaque and the 45 wt.% CaCl_2_/silk film being the most transparent. These findings are consistent with the quantitative results obtained from UV–vis spectroscopy.

Aside from transparency, epidermal bioelectronics also requires high adhesion and efficient air permeability to maintain stable signal recording, and when worn on the skin for prolonged periods, they should not irritate. As of now, the acquisition of commercial medical‐grade adhesives has been instrumental in achieving these properties. However, distinct adhesives tailored to specific applications are necessary for sensitive skin (such as newborn babies or the elderly). Furthermore, despite their effectiveness, the attachment and subsequent peeling off of these adhesives, particularly around hairy regions, result in mild discomfort or pain. On the contrary, the present solution is versatile, as it caters to individuals with sensitive skin and those without, rendering it to be universally applicable. The self‐adhesive property of the silk bioelectronics is demonstrated by quantitatively measuring the peel force with respect to glass (Figure 2g; Figure S11, Supporting Information). The resultant force (≈300 N m^−1^) was comparable to commercial polyimide Kapton tape (≈150 N m^−1^), medical grade adhesives,^[^ ^79^ ^]^ and previously reported skin‐like electronics.^[^ ^80^, ^81^, ^82^, ^83^ ^]^ Remarkably, the silk films are not severely prone to dehydration as the adhesion force dropped only by 25% after leaving the silk films in ambient air for 1 week (Figure 2g blue curve). Additionally, the effects of multiple heating cycles (dehydration) followed by exposure to high humidity (rehydration) were investigated by subjecting 100 µm thick silk films to heating at 75 °C for 1 h, followed by treatment at 60% relative humidity (RH) and 22.6 °C for 30 min, repeated for a total of ten cycles. Weight variations were measured throughout this process, indicating that silk films can effectively regain their water content after ten consecutive dehydration and hydration cycles (Figure S12a, Supporting Information). Furthermore, a 90° peeling test was performed to quantitatively assess the impact of cyclic hydration and dehydration on the adhesivity of the silk films. The results showed minimal variations (12.5%) in adhesive strength between samples subjected to cyclic conditions and those that were not, suggesting that repeated heating and water recovery do not negatively impact the performance of the silk films (Figure S12b, Supporting Information).

In addition, skin‐like bioelectronics should permit the skin‐air circulation of water and oxygen to prevent any dermatological infection or allergy (i.e., they should have high air permeability). To show the breathability of the reported silk films, we analyzed the water vapor transmission rate (WVTR) of  45 wt.% CaCl_2_‐loaded silk films with a thickness of 100 µm (Figure S13, Supporting Information).^[^ ^84^ ^]^ Accordingly, the water was heated to 80 °C, and the weight drop due to the evaporated water molecules escaping through the silk films was measured. The obtained WVTR was 1262.6 g h^−1^ m^−2^, which is comparable to that of human skin with a WVTR of ≈12 g h^−1^ m^−2^.^[^ ^85^ ^]^ To further show the air permeation of the silk substrate in situ, thermal analysis was performed after “wearing” the Silk‐BioE and commercial gel‐based Silver/Silver Chloride (Ag/AgCl) electrodes on the forearm for 5 h, and no temperature variations were observed on the skin region covered with Silk‐BioE and Ag/AgCl electrodes after immediate thermal imaging (Figure S14, Supporting Information). These findings highlight Silk‐BioE as a promising candidate for an irritation‐free electrode, offering extended monitoring periods comparable to those of commercial alternatives.

### 2.3 Printing and Characterization of the Inkjet‐Patterned Silk Bioelectronics

**Figure 3:**
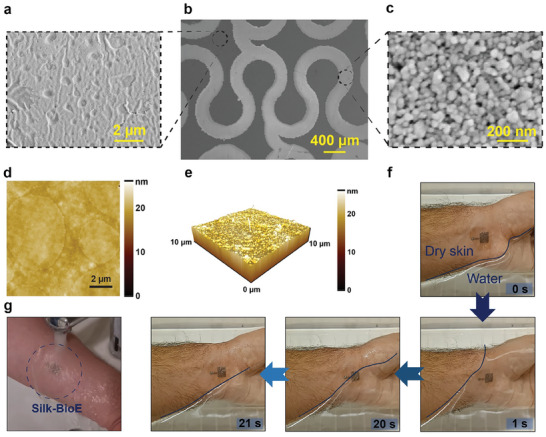
Surface topography and water‐resistant adhesion of the skin‐like silk bioelectronics (Silk‐BioE). SEM images of a) the pristine silk substrate section of the Silk‐BioE, b) Silk‐BioE with inkjet‐printed AgNP patterns, and c) zoom‐in view of the AgNP section of the Silk‐BioE. AFM images showing: d) the surface topology, e) 3D topography of the silk substrate, f) Evaluation of the adhesion quality of Silk‐BioE on skin under water prior to submersion in water, during full submersion of the hand in water, with the hand partially submerged and partially exposed to air, and after removal from the water, and g) Silk‐BioE on skin under running water.

The surface topography of the patterned silk substrate with silver nanoparticle (AgNP) was examined using SEM and atomic force microscopy (AFM). The SEM images depict the serpentine pattern of inkjet‐patterned AgNP on the silk substrate (**Figure** **3**b), with the printed AgNP estimated to have a nanoparticle size of ≈75 nm (Figure 3c). Furthermore, Figure 3a in the SEM image illustrates the surface roughness of the silk electrodes. This observed roughness was compared with the AFM images (Figure 3d,e), revealing a maximum surface roughness of 26.29 nm.

To evaluate the combined effects of body hair and perspiration on Silk‐BioE, artificial sweat was applied to a male participant's densely hairy chest both prior to and following electrode placement, with simultaneous electrocardiography (ECG) recordings. The high‐quality ECG results demonstrated that high humidity conditions, including sweat, and the presence of natural body hair do not negatively impact the device's performance (Movie S2, Supporting Information). Additionally, to quantitatively evaluate the influence of sweat and body hair on the accuracy of physiological signal recordings, ECG data were collected from both dry and sweaty skin areas covered with hair. A comparative analysis of these signals indicated that the ECG from the sweaty, hairy region exhibited an SNR of 18.24 dB, surpassing the SNR of 17.16 dB recorded from the dry, hairy region (Figure S15, Supporting Information). This improvement in signal quality can be attributed to the abundance of sodium and chloride ions in sweat, which enhance the skin‐electrode interface by facilitating electron transfer from the skin to the electrode.^[^ ^86^ ^]^ Consequently, these findings suggest that sweating does not adversely affect signal quality; instead, the ionic composition enhances the quality of recordings from hairy body regions. Moreover, Silk‐BioE retained its original form and adhesive properties despite being immersed in a water bath or after submerged in continuously running sink water (Figure 3f,g and Movie S3, Supporting Information). On the other hand, washing off the skin is possible only by gentle rubbing action on the silk patch underwater (Movie S4, Supporting Information).

**Figure 4:**
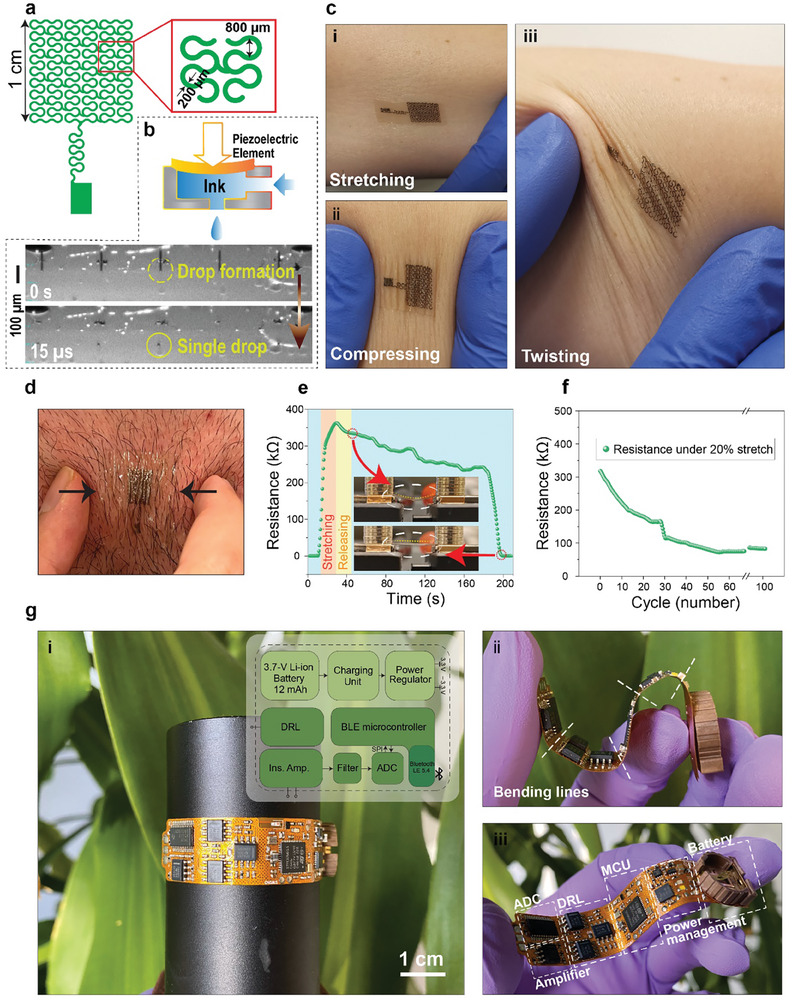
Mechanical properties of the skin‐like silk bioelectronics (Silk‐BioE) and their flexible readout circuitry. a) Geometry of the serpentine design. b) AgNP jetting profile of the inkjet‐printer head. c) Silk‐BioE on the skin under (i) stretching, (ii) compressing, and (iii) twisting motions. d) Feasibility of attaching Silk‐BioE to hairy skin areas. e) Resistance variation of the patch under 20% stretching and releasing tests. f) Resistance variation profile of the patch in 100 times cyclic stretching‐releasing. g) Flexible circuitry (i) on a curved surface (inset: block diagram of the different parts of the circuitry), (ii) folded by hand, and (iii) bent in hand and illustrating the different parts of the system. DRL: driven right leg. BLE: Bluetooth low energy. Ins. Amp.: instrumentation amplifier. ADC: analog to digital converter. SPI: serial peripheral interface.

The Silk‐BioE incorporates inkjet‐printed serpentine designs featuring a 200 µm trace width, an 800 µm circular diameter, and an overall effective surface area of 1 cm^2^ (**Figure** **4**a). Achieving high‐resolution inkjet printing involved optimizing jetting parameters such as piezoelectric jetting voltage, temperature, and frequency. Detailed printing parameters are outlined in the Experimental Section. The measured contact angle of 11° between the AgNP ink and the corona‐treated donor polyethylene terephthalate (PET) substrate (Figure S16, Supporting Information) indicates that the surface energy is sufficiently increased to facilitate the merging of ink droplets on the substrate to form the desired print design. Stylus profilometry indicated a printed AgNP thickness of ≈500 nm on the donor PET substrate (Figure S17, Supporting Information). Following printing, the samples underwent annealing for 20 min at an optimized temperature of 140 °C. This process resulted in a sheet resistance value of 1.24 Ω per square (details shown in Figure S18, Supporting Information). While higher annealing temperatures typically enhance electrical conductivity, the low glass transition temperature of PET substrate at ≈78 °C restricts higher annealing temperatures due to the risk of physical deformation.^[^ ^87^ ^]^

Patterns are susceptible to oxidation due to ambient air which potentially impacts their mechanical and electrical properties. To demonstrate the shelf‐life of inkjet‐printed silver‐based soft electronics, we conducted experiments revealing that low humidity conditions can effectively safeguard the AgNP electrodes from oxidation (Figure S19, Supporting Information). Samples stored in a desiccator exhibited no signs of oxidation or changes in resistance compared to those exposed to ambient air, which displayed a resistance increase of 76.3% after 1 month. Despite the large percent change in resistance, the initial resistance of the printed structures (4.65 Ω) only changes by ≈3.55 Ω in 1 month, bringing the final resistance to 8.2 Ω which is still very conductive for a typical single‐use biopotential electrode. Therefore, we envision that inkjet‐printed, silver‐based soft electronics can still be re‐used for durations in excess of a couple of months for various applications including human vital signs monitoring. Figure 4b illustrates the jetting profile of 5 out of 12 nozzles. It showcases uniform jetting of AgNP ink droplets from the nozzles, exhibiting perfectly rounded shapes without secondary satellites or stretched filaments. The duration for inkjet‐printing a single electrode was estimated to be 5 min.

Conformal attachment of Silk‐BioE in mimicking the skin deformations under stretching, compressing, and twisting is shown in Figure 4c (Also see Movie S5, Supporting Information). In contrast to previously reported epidermal (bio)electronics, which are limited to application on hairless or shaved skin regions,^[^ ^88^ ^]^ our study also demonstrates the conformability of Silk‐BioE on hairy body parts (Figure 4d; Figure S20, Supporting Information). Furthermore, Silk‐BioE provides a highly comfortable peeling and gentle removal experience, even from densely hairy areas of skin. Indeed, unlike traditional commercial electrodes, which often adhere excessively and cause discomfort or pain during hair removal, Silk‐BioE minimizes irritation and ensures a pain‐free application (Movie S6, Supporting Information).

To evaluate the maximum adhesion of Silk‐BioE on hairy regions of the skin under severe humid conditions, such as during perspiration, ECG data was collected from a participant's hairy skin for a continuous duration of 1 h. Artificial sweat was applied to the Silk‐BioE electrodes every 10 min to replicate continuous sweating during intense workouts. The results demonstrated that the SNR values at the beginning (18.12 dB) and at the end (18.08 dB) of the recording showed minimal variation (Figure S21, Supporting Information). These findings indicate that Silk‐BioE is suitable for prolonged use on hairy and perspiring areas of the body without significant degradation in signal quality.

When subjected to a 20% strain, the recorded electrical resistance exhibited a maximum change of 352.5 kΩ (Figure 4d). This change in resistance is attributed to surface cracks in the printed AgNP that emerge under strain. The increased resistance gradually returns to its initial value once the strain is removed, primarily because the transition from plastic to elastic deformation in the silk substrate occurs gradually. The electrical performance of the Silk‐BioE was further evaluated by attaching an electrode integrated with a light‐emitting diode (LED) to a balloon (Figure S22, Supporting Information). A consistent electrical connection maintained the illumination of the LED as the Silk‐BioE underwent stretching or compression with the inflation or deflation of the balloon. Similarly, the electrode with an LED exhibited stable performance on the skin during stretching, twisting, and compressing (Figure S23, Supporting Information). The durability/endurance of the Silk‐BioE was also assessed through cyclic of stretching and releasing at 20% strain for 100 repetitions. Throughout these cycles, the electrode resistance decreased from ≈300 to ≈80 kΩ when subjected to 20% stretch.

The integration of Silk‐BioE with our flexible readout circuitry having a 15 × 70 mm footprint forms a compact system capable of continuously recording various electrophysiological biosignals, such as non‐invasive biopotentials. The sensing circuitry (Figure 4f) comprises an instrumentation amplifier (INA) with a gain of 180, followed by a low‐pass active filter (LPF) set at a 1 kHz cut‐off frequency without gain (Figure S24, Supporting Information). The analog signal then enters a 32‐bit resolution delta‐sigma analog‐to‐digital converter (ADC) operating at a sampling rate of 360 samples per second for further processing. Power is supplied by a rechargeable 12 mAh lithium‐ion battery, managed by the battery management unit for on‐board recharging. A Bluetooth low energy (BLE) system‐on‐chip (SoC) facilitates the reception of digitized data from the ADC and its wireless transmission to a nearby smartphone. The individual components of the readout circuit were assembled on a flexible printed circuit board (FPCB), which can bend and conform to various curved surfaces for seamless integration into the human body and the wearable Silk‐BioE. The electrical performance of the flexible device remains consistent even under mechanical bending exceeding 90°, surpassing the natural deformations of the body regions of interest, such as the chest, neck, and forearm.

### 2.4 Silk‐BioE Application in ECG, EMG, EOG, and EEG Recording

**Figure 5:**
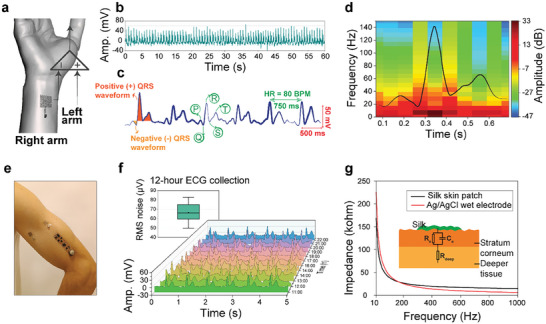
a) ECG signal collection using skin‐like silk bioelectronics. b) P‐QRS‐T complex identification with an estimated heart rate from the recorded ECG signal. c) Power analysis of the recorded ECG signal. d) Schematic illustration of the Silk‐BioE placement for ECG recording. e) Silk‐BioE integrated with the flexible signal acquisition unit. f) Extended ECG monitoring using the Silk‐BioE over 12 h. g) Comparison of skin‐electrode impedance between “soft” Silk‐BioE and commercial “wet” Ag/AgCl electrodes where the inset shows an illustrative electrical model of the impedance.

The Silk‐BioE can capture electrical biopotential signals throughout human skin emanating from physiological processes. To demonstrate its functionality, we collected ECG signals in a standard lead‐I configuration by placing the Silk‐BioE on the left and right inner wrist as differential electrodes while the ground electrode was positioned on the left arm (**Figure** **5**a). These electrodes, characterized by their high elasticity, proved ideal for acquiring high‐quality ECG signals showcasing distinguishable P‐QRS‐T segments (Figure 5b,c). The fidelity of these signals allows for diagnosing cardiovascular conditions like ischemic heart disease and myocardial infarction^[^ ^89^ ^]^ and estimating heart rate in real‐time. The peak‐to‐peak voltage of the recorded ECG signals measured ≈60 mV, with the gain of the flexible electronic circuitry set at 180, and the Welch spectral density estimation indicates a power value of 33 dB for the R‐peak within the frequency range of 0–150 Hz (Figure 5d).

In addition, Figure 5e illustrates that the Silk‐BioE, along with the flexible board, serves as an effective soft electrode for single‐arm ECG monitoring (Figure S25, Supporting Information). Furthermore, a comparative assessment was conducted to evaluate the performance of our “soft” silk electrodes in parallel with commercial “wet” Ag/AgCl electrodes for simultaneous ECG signal measurement using a commercial open‐source Open‐BCI data acquisition unit (Figure S26, Supporting Information). The recorded signals from both the Silk‐BioE and Ag/AgCl electrodes exhibited an almost excellent Pearson's correlation ratio of 99.4% and similar SNR values of 9.09 dB (Silk‐BioE) and 10.08 dB (Ag/AgCl), affirming the high fidelity of the Silk‐BioE in capturing ECG signals.

The superior self‐adhesiveness of the silk electronics ensures a conformal attachment to the skin, enabling the recording of ECG signals for up to 12 h without any compromise in signal quality. Throughout this duration, the participant was able to engage in routine activities such as sleeping, eating, and performing physical tasks like laboratory work. To ensure the accuracy of the ECG recordings and verify that the adhesivity of the silk had not degraded during the 12 h, ECG signals were recorded at 1‐h intervals in a stationary position. Figure 5f presents the 5‐s snapshots of these recordings, providing a total of 12 distinct ECG signals over the 12‐h period. The RMS noise values fluctuated between 50 and 82 µV, with an average value of 66.5 µV. This highlights the minimal influence of body movements on the adhesiveness of the Silk‐BioE, thereby affirming the consistent performance of the electrode.

Interestingly, the self‐adhesive Silk‐BioE exhibits an even lower skin‐electrode impedance than commercial electrodes equipped with adhesive backing within the 10–1000 Hz range (Figure 5g). In an experimental setup where both the commercial Ag/AgCl and Silk‐BioE electrodes were placed on the forearm with a 5 cm spacing,^[^ ^90^ ^]^ impedance measurements at 10 Hz showed values of 450.67 kΩ for the Ag/AgCl electrodes and 337.16 kΩ for the Silk‐BioE. The decreased impedance of the Silk‐BioE is attributed to its exceptional elasticity and robust adhesion at the interface between silk and skin. These material properties facilitate minimal air gaps between the skin and the electrode, reducing the capacitive effect and consequently lowering impedance values.^[^ ^63^ ^]^ Additionally, to investigate the effect of high humidity conditions on the performance of Silk‐BioE, skin‐electrode impedance was measured at two relative humidity levels of 45% and 80% RH. The results (Figure S27, Supporting Information) indicate that high ambient humidity is associated with an improvement in skin‐electrode impedance, with a decrease from 375.28 kΩ at 45% RH to 139.16 kΩ at 80% RH (at 10 Hz). This improvement is primarily attributed to the increased elasticity of silk in high humidity, which enhances the interface between the skin and the electrode, potentially leading to improved signal quality.

**Figure 6:**
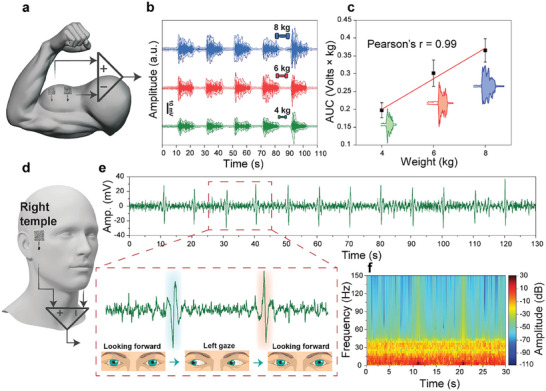
EMG and EOG recording with Silk‐BioE: a) the placement of Silk‐BioE electrodes on the bicep for EMG monitoring. b) EMG signals were recorded in real‐time during the contraction and relaxation phases of the bicep muscle in lifting various dumbbells. c) Calibration curve linking the area under the curve (AUC) of EMG signals to the weight of different dumbbell weights, which can be used to quantify exercise intensity in training or in notifying/predicting muscle fatigue. d) Electrode placement for EOG recording. e) EOG acquisition by Silk‐BioE during left and right eye rotations can be used within activity recognition and mobile human‐machine interaction (HMI). f) Analysis of the EOG power spectrum density.

The unique properties of the Silk‐BioE, including self‐adhesiveness, stretchability, and breathability, render it exceptionally suitable for assessing the electrical activity of the bicep brachii through recording electromyography (EMG) signals. EMG signal collection typically targets specific muscle groups during consistent physical activity, which can induce considerable skin deformation and motion artifacts. Choosing an inappropriate electrode under such conditions may compromise signal quality and data loss. Self‐adhesive and stretchable electrodes prove advantageous for EMG signal recording as they mimic severe skin deformations and enhance signal fidelity. Additionally, the skin's increased sweat production during physical activity necessitates breathable materials to prevent potential localized skin trauma.^[^ ^91^ ^]^ To record EMG signals, a pair of Silk‐BioE electrodes were positioned 2 cm apart on the right bicep (**Figure** **6**a). The participant performed a series of exercises involving lifting dumbbells of varying weights and maintaining muscle contraction for 10 s, followed by a 10‐s relaxation period. This experiment was repeated using dumbbells weighing 4, 6, and 8 kg. The recorded EMG signal amplitudes during muscle contraction were ≈10, 20, and 30 mV for the 4, 6, and 8 kg weights, respectively (Figure 6b). Furthermore, the intensity of the measured signal demonstrated a linear correlation with the dumbbell weight, exhibiting a Pearson correlation coefficient (r) value of 0.99 (Figure 6c).

Furthermore, the application of Silk‐BioE extends to collecting electrooculography (EOG) signals. In our setup, differential electrodes were positioned on the right and left temples (Figure 6d), and as the participant shifted their gaze between right and left orientations, a distinct alteration in voltage amplitude in the cornea‐retinal potential, spanning from the front to the back of the eye, was observed. This particular change bears significance in identifying ocular disorders, facilitating early diagnosis of macular degeneration, and fostering advancements in human‐computer interaction (HCI).^[^ ^92^, ^93^ ^]^ The recordings illustrated in Figure 6e depict the EOG signals acquired through Silk‐BioE and flexible circuitry while the participant executed swift horizontal eye movements at regular intervals. The experimental protocol involved the participant looking forward, then shifting their gaze successively to the left, returning to the center, and subsequently to the right, each position held for 10 s. The distinctive signal profiles during left and right eye movements exhibited fluctuations within the approximate range of −30 to 30 mV. Analysis using Welch's Fourier transform unveiled power magnitudes in the recorded signals oscillating between −110 and 30 dB (Figure 6f).

**Figure 7:**
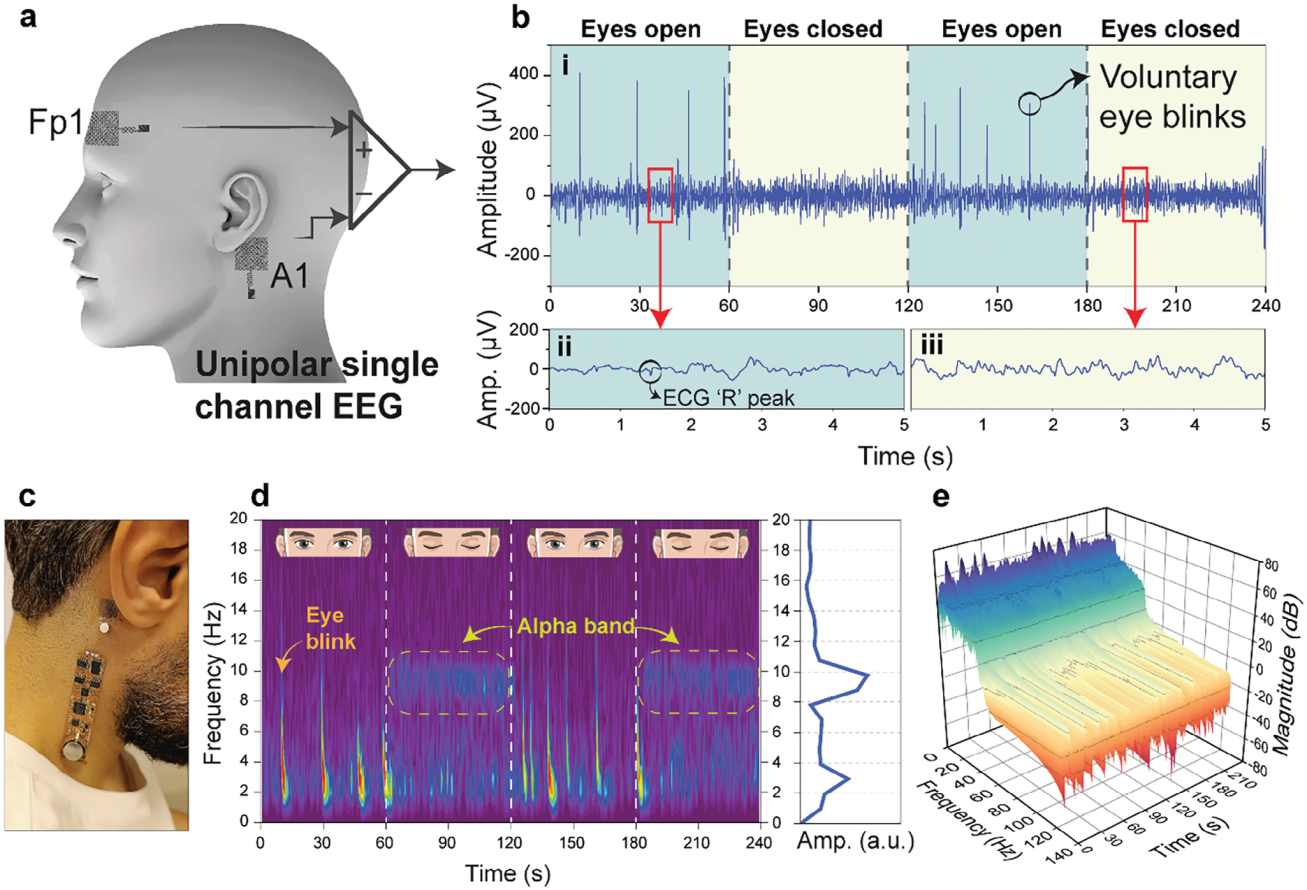
EEG Signal Acquisition using Silk‐BioE: a) Schematic representation illustrating the placement of Silk‐BioE electrodes on Fp1 and A1 for EEG acquisition. b) Time‐domain EEG signals were captured during both the open and closed‐eye states of the participant. Insets emphasize frequency variations observed during the alternating eye movements. c) Integration depiction of Silk‐BioE with the flexible signal acquisition board for EEG signal collection. d) Frequency‐domain representation of EEG signals highlighting the emergence of alpha‐wave frequencies during closed‐eye states. e) Power spectrogram showcasing the spectral analysis of the EEG recordings.

Moreover, we investigated single‐channel electroencephalography (EEG) signal recording with the Silk‐BioE. Electrodes were positioned in a configuration adhering to the universally standardized 10–20 system, specifically on Fp1 and A1 (**Figure** **7**a). While all the previous biopotentials (ECG, EMG, and EOG) typically exhibit voltage amplitudes in the milli‐volt range, EEG signals fall within the micro‐volt range, rendering them particularly susceptible to noise. Therefore, the performance of both electrodes and the electronic signal acquisition unit is critical in meeting the stringent requirements for EEG signal collection. To validate the accuracy of the recorded EEG signal, we assessed the generation of alpha‐band frequencies. The alpha band, spanning from 8 to 12 Hz,^[^ ^94^ ^]^ typically manifests during participant relaxation, particularly when their eyes are closed. As such, the participant was instructed to open and close their eyes every minute alternately. Time‐domain EEG signals recorded using Silk‐BioE and the flexible signal acquisition unit are depicted in Figure 7b. In part (i) of Figure 7b, the EEG signals manifest distinct sharp spikes associated with voluntary eye blinks (EOG), marked by an approximate amplitude of 400 µV. However, during the eye closure, these spikes are substituted by the rapid eye movement state (REM). Higher‐frequency alpha‐band generation is also discernible in the time‐domain results (Figure 7b (ii,iii)). On the other hand, the interesting observation of the presence of ECG (R‐peak) within the recorded EEG signals underscores the high performance of Silk‐BioE in capturing high‐resolution biosignals and low‐noise recording where even the forehead ECG signals are distinguished. Indeed, the short‐time Fourier transform (STFT) illustrates the occurrence of alpha‐band frequencies (7–11 Hz) when eyes are closed (Figure 7d). Furthermore, Welch's Fourier transform analysis depicts a power magnitude of 80 dB within the recorded EEG signals. To further evaluate the performance of the proposed Silk‐BioE and electronic circuitry in EEG signal recording, participants were tasked with maintaining relaxed open‐eye states for 60 s, followed by closing their eyes and engaging in focused mental arithmetic exercises with intense concentration (e.g., such as calculating the product of 12–135). Results reveal the emergence of beta frequencies (15–18 Hz) during focused closed‐eye states (Figure S28, Supporting Information).

## 3 Discussion

This study introduces a tailored solution for epidermal bioelectronics by unifying all the essential features that skin‐interfaced skin‐electronics must possess and do so by reformulating of the green synthesis of an ancient material—protein‐based natural silk derived from *Bombyx mori* cocoons. Departing from the conventional silk synthesis approaches, this refined formulation harmonizes the formerly disjointed features yet essential for epidermal electrodes, such as self‐adhesiveness, breathability, biodegradability, and elasticity. Consequently, our soft electronics effectively merge the advantages of both wet and dry electrodes, creating a conformal skin/electrode interface akin to wet electrodes while ensuring long‐term, irritation‐free use characteristic of dry electrodes. This achievement is attributed solely to the merger of high self‐adhesiveness (300 N m^−1^ on glass) and good elasticity (Young's Modulus of 5.1 kPa and >600% stretchability) as well as near excellent breathability (1262.6 g h^−1^ m^−2^) of our soft silk electronics which facilitates high fidelity in biosignal recording comparable to the “gold standard” wet Ag/AgCl electrodes.

Furthermore, our technology demonstrates remarkable self‐adhesion, ensuring a secure attachment to the skin even in conditions, such as exposure to running water or immersion in a water bath. This resilience not only enhances the practicality of our electrodes for everyday use but also underscores their reliability for long‐term physiological monitoring. A key advantage of Silk‐BioE is its effective adherence to hairy regions of the body, even under conditions of significant perspiration—circumstances that typically compromise the performance of conventional skin electronics. While many devices are designed to cater to either hairy areas^[^ ^95^ ^]^ or perspiration,^[^ ^96^ ^]^ few successfully address both simultaneously. Strong adhesives are often required for secure attachment to hairy regions; however, they frequently lead to painful removal. In contrast, our technology uniquely integrates robust adhesion with exceptional elasticity, ensuring a secure fit while facilitating smooth and comfortable removal.

Shifting to another significant innovation, our practical patterning method effectively addresses the challenges associated with Ca^2^⁺‐modified silk films. The high adhesiveness of this material renders traditional contact patterning techniques, including those requiring masks, impractical. Additionally, its non‐hydrophilic nature complicates surface treatments, making non‐contact deposition methods unfeasible. Moreover, the material's sensitivity to chemical agents alters the protein structure, precluding the use of lithographic processes that involve multiple chemicals.^[^ ^97^ ^]^ Our approach addresses these limitations through a straightforward transfer printing technique, facilitating precise and high‐quality patterning.

In addition to its superior performance characteristics, Silk‐BioE is biodegradable, a critical factor in addressing the environmental concerns associated with electronic waste. Unlike synthetic polymers, which persist in the environment and cause pollution, our Silk‐BioE decomposes naturally within just 2 days, thereby alleviating the burden of e‐waste.

By simultaneously benchmarking our soft electrodes with wet commercial medical‐grade electrodes in lead‐I ECG recording, an almost excellent Pearson's correlation ratio of 99.4% was achieved, showcasing the feasibility of medical‐grade P‐QRS‐T complex analysis using our soft electrodes. Similarly, SNR values of 9.09 dB (Silk‐BioE) compared to 10.08 dB (Ag/AgCl) were observed, verifying the capability for high‐accuracy biopotential measurements by the developed soft electronics. On the other hand, unlike the gold standard electrodes, our inkjet‐printed 200 µm resolution fine patterned solution exhibits high transparency (>95%) as well as an irritation‐ and pain‐free operation, devoid of even mild discomfort during removal, rendering them nearly imperceptible during wear, which align seamlessly with the “wear and forget” concept of next generation of in situ continuous personalized health monitoring devices.

Furthermore, our research demonstrates the extensive capability of our soft electronics in detecting various human electrophysiological signals using identical electrodes and hardware, with the only variation being the placement of the electrodes. This encompasses the recording not only of neural activities in the hundreds of micro‐volt range (EEG) and milli‐volt ocular activities (EOG), but also, notably, it can capture cardiac activities in the tens of micro‐volt range from EEG signals. This ability to detect such minute signals underscores the superior performance of our electrodes and hardware, further demonstrating their potential for diverse applications in both research and clinical settings. Furthermore, our single‐lead EEG recording analysis extends beyond acquiring alpha waves. It also encompasses the recording of beta EEG waves, which is associated with deep meditation or mental arithmetic calculations rarely reported in the context of single‐channel EEG.

In light of the broader implications of our study, it is noteworthy that silk composites with conductive fillers have been previously explored for human electrophysiological signals monitoring.^[^ ^41^, ^98^ ^]^ However, they face fine‐tuning challenges due to their mechanical properties and limited transparency. Similarly, silk films with deposited conductive materials have been studied^[^ ^39^, ^51^, ^99^, ^100^, ^101^, ^102^, ^103^ ^]^ yet these efforts do not fully capitalize on the unique characteristics of silk, meticulously tuned and optimized to address the existing gaps in skin‐electronic materials. Additionally, the precise patterning of silk films remains a challenge in these reported works due to limitations in fabrication methods. **Table** **1** provides a quantitative comparison between Silk‐BioE and previously reported silk‐based wearable electronics for human physiological sensing.

**Table 1:** State‐of‐the‐art in silk‐based skin‐interfaced electrophysiological signals monitoring. Reproduced with permission.^[^ ^100^ ^]^ Copyright 2022, Elsevier. Reproduced with permission.^[^ ^98^ ^]^ Copyright 2021, American Chemical Society. Reproduced with permission.^[^ ^101^ ^]^ Copyright 2024, John Wiley and Sons. Reproduced with permission.^[^ ^51^ ^]^ Copyright 2020, American Chemical Society. Reproduced with permission.^[^ ^102^ ^]^ Copyright 2024, John Wiley and Sons. Reproduced with permission.^[^ ^41^ ^]^ Copyright 2024, John Wiley and Sons. Reproduced with permission.^[^ ^39^ ^]^ Copyright 2024, John Wiley and Sons. Reproduced with permission.^[^ ^103^ ^]^ Copyright 2018, American Chemical Society.

Refs.		Conductive material	Deposition technique	Patterning resolution	ECG	EMG	EOG	EEG	Hairy‐skin adaptability	Breathbility (g h^−1^ m^−2^)	Stretchability (%)	Youngs modulus (MPa)	Biodegradability (days)	Transparency (%)	Adhesiveness (N m^−1^)	Portable sensing system
*Chm. Eng*. **2022** ^[^ ^100^ ^]^	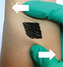	Ppy	Dip‐coating	cm‐scale	**✓**	**✓**	✗	✗	NO	Not reported	> 100	79.62	≈ 15	**✗**	13.5 (on glass)	**✗**
*ACS Nano* **2021** ^[^ ^98^ ^]^	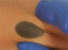	PEDOT:PSS	Blend	cm‐scale	**✓**	**✓**	✗	✗	NO	≈ 117 (37 °)	> 250	≈ 190	Not reported	**✗**	Not reported	**✗**
*Adv. Mat*. **2021** ^[^ ^101^ ^]^	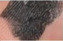	CNT	Brushed	cm‐scale	**✓**	**✓**	✗	✗	NO	Not reported	≈ 35	Not reported	Not reported	**✗**	Not reported	**✗**
*ACS Matt. Lett*. **2020** ^[^ ^51^ ^]^	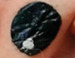	Ppy	Interfacial polymerizatio	cm‐scale	**✓**	**✓**	**✓**	**✓**	NO	Not reported	≈ 300	236 (≈ 53% RH)	Not reported	**✗**	40 (94.6% RH, human skin)	**✗**
*Adv. Func*. **2020** ^[^ ^102^ ^]^	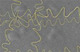	Au	Lithography	nm‐scale	✗	**✓**	✗	✗	NO	Not reported	Not reported	0.134	Not reported	Not reported	Not reported	**✗**
*Adv. Func*. **2019** ^[^ ^41^ ^]^	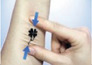	Graphene	Blend	mm‐scale	**✓**	✗	✗	✗	NO	Not reported	Not reported	Not reported	Not reported	**✗**	Not reported	**✗**
*Adv. Mat*. **2018** ^[^ ^39^ ^]^	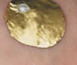	Au	Thermal evaporation	cm‐scale	✗	**✓**	✗	✗	NO	Not reported	> 400	1.66 (50% RH)	Not reported	**✗**	90 (human skin)	**✗**
*ACS Nano* **2018** ^[^ ^103^ ^]^	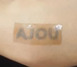	AgNWs	Stencil‐print	cm‐scale	**✓**	✗	✗	✗	NO	71.6 ‐ ‐168.5 (50 °C)	Not reported	0.010 (50% RH)	Not reported	90	10 (porcine skin)	**✗**
**This work**	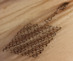	AgNPs	Inkjet printing	200 µm	**✓**	**✓**	**✓**	**✓**	YES	1262.6 (80 °C)	> 600	0.0051 (60% RH)	2	> 95	≈ 300 (on glass)	**✓**

Moreover, silk‐based skin‐like bioelectronics stand distinct from synthesized polymer‐based materials. Indeed, other skin‐like electronics with adhesive properties often require a secondary separate adhesive polymer layer,^[^ ^104^, ^105^, ^106^, ^107^, ^108^, ^109^, ^110^, ^111^ ^]^ complicating the fabrication process and adding cost and material usage. Fabrication processes based on microfabrication techniques being resource‐intensive and necessitating a well‐regulated environment, lead to substantial costs and intricacy in fabrication. This, in turn, results in high‐priced final products, thereby restricting their widespread applicability.^[^ ^107^, ^108^, ^110^, ^112^, ^113^ ^]^ Integrating conductive fillers into polymer matrices limits proper permeability and optical transparency.^[^ ^17^, ^80^, ^95^ ^]^ Importantly, synthetic polymers and plastics lack natural decomposition, creating e‐waste and environmental concerns.^[^ ^22^, ^81^ ^]^ Table S2 (Supporting Information) provides a qualitative comparison between silk‐based skin‐like bioelectronics and various previously reported skin‐like wearable bioelectronics, highlighting its unique advantages.

Therefore, this study presents an all‐in‐one solution, poised to propel the next generation of epidermal bioelectronics applications, transcending healthcare, wellness, and fitness domains, and maybe introduce novel electrophysiological sensory inputs for emerging augmented and virtual reality platforms whose electrode placement configuration always includes the forehead.

## 4 Experimental Section

### Silk Solution Preparation

Natural *Bombyx mori* cocoons, ≈4 cm long (purchased from a local vendor, Istanbul, Turkey), were sectioned into ≈1 cm^2^ segments using stainless‐steel scissors. To initiate the degumming process—the process to remove the sericin protein, a sodium carbonate solution (40 mmol l^−1^) was prepared by dissolving Na_2_CO_3_ powder (8.48 g) (Sigma–Aldrich, USA) in deionized water (2‐liter). This solution was then elevated to a temperature of 100 °C under an aluminum foil seal in a 2‐liter glass beaker. Subsequently, the prepared cocoon segments (10 g) were introduced into the boiling Na_2_CO_3_ solution and subjected to constant stirring for 45 min. After this treatment, cocoons were carefully withdrawn from the solution and thoroughly rinsed with deionized water. Excess water was removed by squeezing the cocoons. The treated cocoon segments were then rinsed in deionized water for 20 min to clear the residual sodium carbonate and sericin fully, and this was repeated three times. The treated cocoon segments were left to air dry overnight on aluminum foil within a fume hood. To prepare the 45 wt.% CaCl_2_/silk solution, calcium chloride (CaCl_2_, Sigma–Aldrich, USA) (1.35 g) was mixed with formic acid (HCOOH, Sigma–Aldrich, USA) (20 g). This mixture was stirred until no visible CaCl_2_ particles remained discernible. Following the dissolution of CaCl_2_, previously degummed cocoons was gradually introduced into the solution (3 g) while the mixture was stirred at 700 rpm for 1 h at room temperature. Subsequently, the solution was transferred into an ultrasonication bath for 30 min at 45 °C to dissolve silk fibers further.

### Formation of Skin‐Like Silk Bioelectronics

First, the 130 µm polyethylene terephthalate (PET) films were cleaned using isopropanol alcohol and deionized water and then dried using a nitrogen gun. Next, the surface wettability of the films was improved through a 1‐min corona treatment to enhance ink adhesion to the plastic surfaces (BD‐20AC, Electro‐Technic Products, USA). The inkjet printing of silver nanoparticles ink (AgNP, JS‐A102A, Novacentrix, USA) was carried out employing an inkjet printer (DMP‐2850, Fujifilm, USA) equipped with a loaded cartridge (Samba Dimatix, Fujifilm, USA), which featured twelve nozzles capable of jetting a minimum drop volume of 2.4 pL. A drop spacing of 15 µm was configured to ensure optimal print quality and electrical conductivity. The jetting process was executed under the following conditions: nozzle voltage of 30 V, nozzle temperature of 28 °C, and a jetting frequency of 10 kHz. The default Fujifilm “Model Fluid” waveform was applied to the piezoelectric elements, resulting in a calculated drop velocity of 6.6 m s^−1^, and all the patterns were printed in a single printing cycle. Subsequently, the printed AgNP patterns were annealed at 140 °C for 20 min. Later, the as‐printed serpentine AgNP network was transferred onto silk films by drop‐casting silk solution onto the newly patterned PET substrates. It was experimentally found that to achieve a silk film thickness of 100 µm, a volume of 50 µL cm^−2^ of the silk solution was required to be drop‐casted. After applying the silk solution onto the donor substrates, each sample underwent vacuum desiccation to ensure no visible air bubbles remained within the silk solution. Finally, the samples were left to dry for 20 h within a fume hood, allowing for the complete evaporation of formic acid. Next, a post‐baking step was performed to detach the PET substrate from the silk/AgNP layers and enhance the peeling process for 45 wt.% CaCl_2_/silk films with a thickness of 100 µm. This thermal treatment involved heating the samples at 75 °C for 15 min in an oven. These parameters were empirically optimized to achieve the most effective peeling process, which ensures that the force required to separate the elastic silk from the donor substrate does not push the silk into its plastic deformation region. Subsequently, the separated silk films, now bearing AgNP patterns but still rigid, were subjected to a humidity treatment for 15 min within a controlled humidity chamber at a moisture level of 70% RH. This treatment restored the silk substrate to its initial soft and stretchable state, forming the skin‐like silk bioelectronic patch.

### Flexible Readout Circuitry

A compact and lightweight circuitry, measuring 70 × 15 mm in dimensions and weighing 10 g, was designed on a flexible 0.1 mm‐thick polyimide substrate to facilitate low‐power recording of multimodal biopotential signals with data transmission capability. At the heart of it resides a 32‐bit SOC (STM32WB55, STMicroelectronics, Switzerland), which incorporates an integrated BLE module. The custom‐designed analog signal conditioning circuitry includes an instrumentation amplifier, an active band‐pass filter, and a driven‐right‐leg (DRL) circuit. Upon acquiring signals and their initial analog filtering, the recorded data was digitized by a 32‐bit analog‐to‐digital converter (ADC, ADS1281, Texas Instruments, USA). This ADC operates at a sampling rate of 360 samples per second. With a maximum power consumption of 76 milliwatts, the electronic board can operate for 30 min using a 12 mAh rechargeable battery. By incorporating a recharging unit on the board, the battery can be recharged directly, eliminating the need for removal and external charging. Two small magnets featuring conductive surface coatings were strategically employed to sandwich silk electrodes with the FPCB pads to connect the silk bioelectronics with the flexible wireless data acquisition unit. These magnets exert pressure on the copper wires, ensuring a secure and reliable connection with the inkjet‐patterned silver pads (Figure S25, Supporting information).

### Biopotential Recording

Biopotential recording studies detailed in section 2.4 were performed on voluntary participants with their informed consent by adhering to the guidelines of the Helsinki Declaration of 1964, as revised in 2013 and approved by the ethics committee of Sabanci University (FENS‐2020‐48).

### Statistical Analysis

The data processing was conducted using Origin (OriginLab Corporation, MA, USA) for XRD, Raman spectra, and transmission data. Initially, the baseline of the collected XRD data was removed. Subsequently, both the collected transmission data and XRD data underwent smoothing, which was performed utilizing the Loess method with a smoothing span set at 0.1. Regarding Raman spectra, the data underwent a series of preprocessing steps. Initially, an asymmetric least‐square fit was applied to each spectrum to effectively subtract the baseline, mitigating the influence of autofluorescence. Following this, spectrum smoothing was achieved using the Savitzky–Golay filter. For the collected biopotential signals, offline processing was carried out in MATLAB. The initial step involved the removal of signal baselines. Subsequently, a notch filter with a quality factor of 35 was applied to eliminate the 50 Hz powerline noise. Depending on the type of signal collected, a band‐pass filter specific to that signal type was employed. Finally, a moving average filter was applied to further enhance the signal quality. The Root Mean Square (RMS) was used in the calculation of the SNR for the ECG signal following the equation below:

(1) $$SNR=20\times \log\left(\frac{RMS\left(signal\right)}{RMS\left(noise\right)}\right)$$

## Conflict of Interest

The authors declare no conflict of interest.

## Author Contributions

S.S.M. conceptualized, curated data, conducted formal analysis, investigation, and methodology, developed software, validated, visualized, and wrote the original draft, as well as reviewed and edited the writing. A.G. conceptualized, performed formal analysis, investigation, and methodology, validated, provided resources, wrote the original draft, and reviewed and edited the writing. M.U. conceptualized, conducted investigation, and reviewed and edited the writing. B.A.K. conducted investigation, methodology, and reviewed and edited the writing. F.S.I. conducted investigation, methodology, and reviewed and edited the writing. M.K.Y. conceptualized, performed formal analysis, investigation, and methodology, acquired funding, administered the project, provided resources, supervised, validated, and reviewed and edited the writing.

## Supporting information



Supporting Information

Supplemental Movie 1

Supplemental Movie 2

Supplemental Movie 3

Supplemental Movie 4

Supplemental Movie 5

Supplemental Movie 6

## Data Availability

All the data gathered to reach the conclusions stated in this research was made available in the body of the text or the supporting information. However, if there are further requests, the authors will happily provide them upon request.
