# Supplementary material for: Highly Self‐Adhesive and Biodegradable Silk Bioelectronics for All‐In‐One Imperceptible Long‐Term Electrophysiological Biosignals Monitoring
Source: Adv Sci (Weinh). 2025 Jan 10;12(8):2405988. doi: 10.1002/advs.202405988 (PMC11848544; doi:10.1002/advs.202405988)
Supplement: Supplementary file 1 — Supporting Information [file ADVS-12-2405988-s005.pdf]

ADVANCED SCIENCE
Open Access

Supporting Information

for *Adv. Sci.*, DOI 10.1002/advs.202405988

Highly Self-Adhesive and Biodegradable Silk Bioelectronics for All-In-One Imperceptible Long-Term Electrophysiological Biosignals Monitoring

*Seyed Sajjad Mirbakht, Ata Golparvar, Muhammad Umar, Burcu Arman Kuzubasoglu, Farid Sayar Irani and Murat Kaya Yapici**

Supporting Information

Highly Self-adhesive and Biodegradable Silk Bioelectronics for All-in-one Imperceptible Long-term Electrophysiological Biosignals Monitoring

S. Sajjad Mirbakht [1,2], Ata Golparvar [2,3], Muhammad Umar [1,2,4], Burcu Arman Kuzubasoglu [1,2,4], Farid Sayar Irani [1,2,4], Murat Kaya Yapici [1,2,4,5]*

S. S. Mirbakht, M. Umar, B. A. Kuzubasoglu, F. S. Irani, M. K. Yapici

[1] Faculty of Engineering and Natural Sciences, Sabanci University, 34956 Istanbul, Türkiye.

S. S. Mirbakht, A. Golparvar, B. A. Kuzubasoglu, M. Umar, F. S. Irani, M. K. Yapici

[2] Sabanci University Micro/Nano Devices and Systems Lab (SU-MEMS), Sabanci University, 34956 Istanbul, Türkiye.

A. Golparvar

[3] ICLab, École Polytechnique Fédérale de Lausanne (EPFL), 2002 Neuchâtel, Switzerland.

M. Umar, B. A. Kuzubasoglu, F. S. Irani, M. K. Yapici

[4] Sabanci University SUNUM Nanotechnology Research Center, 34956 Istanbul, Türkiye.

M. K. Yapici

[5] Department of Electrical Engineering, University of Washington, 98195 Seattle, USA.

[*] Corresponding author E-mail: murat.yapici@sabanciuniv.edu

Table of Contents

- **Experimental Section**
  - Materials Characterization
  - Mechanical Measurements
  - Sample Characterization
  - Biodegradability Experiment
  - Water Vapor Transmission Rate (WVTR)
  - Measurement of Adhesion Strength Using 90° Peeling Test
  - Skin-Electrode Impedance Analysis
  - Sample prepration
  - Flexible and Wireless Signal Aquisition Unit Design
  - Humidity Adjustments
  - Thermal Analysis
- **Supplementary Figures**
  - Figure S1: Process of degumming silk cocoons and silk solution preparation.
  - Figure S2: Impact of transfer printing on the integrity of printed AgNP patterns.
  - Figure S3: Stress-strain curves for silk films modified with varying concentrations of $CaCl_2$, ranging from 5 wt% to 50 wt%.
  - Figure S4: Stress-strain test of 45 wt% $CaCl_2$/silk films under 60% RH humidity.
  - Figure S5: Surface topography of PDMS skin-replica.
  - Figure S6: The X-ray diffraction (XRD) analysis of 5wt% and 45 wt% $CaCl_2$/silk films.
  - Figure S7: Biodegradability analysis of silk films in soil.
  - Figure S8: Water solubility analysis of silk films.
  - Figure S9: Surface wettability of silk films with varying $CaCl_2$/silk ratios.
  - Figure S10: Optical transparency characterization of silk films with varying $CaCl_2$ concentrations.
  - Figure S11: Adhesion strength measurement using 90° peeling test with respect to dry glass.
  - Figure S12. The influence of repeated heating and humidity on silk film adhesive properties.
  - Figure S13: Water Vapor Transmission Rate (WVTR) of silk films.
  - Figure S14**:** Thermal characterization of forearm skin following 5-hour application of commercial Ag/AgCl electrodes and Silk-BioE.

- Figure S15: Influence of ionic composition of sweat on ECG recordings from hairy regions.
- Figure S16: Impact of corona treatment on the surface wettability of polyethylene terephthalate (PET) substrates, optimized for inkjet-printing of silver nanoparticle (AgNP) ink.
- Figure S17: Profilometer results for a PET substrate with a single cycle of inkjet-printed AgNP.
- Figure S18: The impact of annealing temperature on the sheet resistance of inkjet-printed AgNP.
- Figure S19: Impact of ambient air on the oxidation of inkjet-printed AgNP samples.
- Figure S20: Feasibility of attaching the Silk-BioE to hairy skin areas due to its superior self-adhesiveness.
- Figure S21. Performance assessment of Silk-BioE on hairy skin during prolonged perspiration.
- Figure S22: Electrical performance of Silk-BioE under conditions of stretching and compression attached on a balloon.
- Figure S23: Electrical performance of an LED-integrated Silk-BioE applied to skin under various skin deformations.
- Figure S24: Design of the analog front end and PCB for the flexible, wireless biopotential signal acquisition unit.
- Figure S25: Single-arm ECG signal acquisition using Silk-BioE and flexible biopotential unit.
- Figure S26: Simultaneous ECG recording using Silk-BioE and Ag/AgCl electrodes.
- Figure S27. Effect of ambient humidity on the skin-electrode impedance of Silk-BioE at 45% and 80% RH.
- Figure S28: Fast Fourier Transform spectroscopy of EEG signals recorded using Silk-BioE, aimed at investigating beta-band frequencies during deep meditation phases.
- Figure S29: Connection between Silk-BioE and flexible signal acquisition unit.

- **Supplementary Tables**

- Table S1: Raman shift location and assignment of Silk Protein.
- Table S2: Qualitative comparison between Silk-BioE and various previously reported non-silk-based skin electronics for human physiological sensing.
- Table S3: Inventory of functional components utilized in the construction of the flexible, wireless biopotential signal acquisition unit.

- **Supplementary Movies**
  - Movie S1: Biodegradability of silk substrates in soil.
  - Movie S2: Excellent self-adhesiveness and robust performance of Silk-BioE in recording electrocardiography signals (ECG) on hairy regions of the body and under severe perspiration.
  - Movie S3: Silk-BioE on skin under running water.
  - Movie S4: Removal of Silk-BioE from the skin through a simple washing process.
  - Movie S5: Silk-BioE on skin, undergoing various phases of compression, stretching, and twisting.
  - Movie S6: Evaluation of removal comfort: Silk-BioE vs. commercial Ag/AgCl electrodes on hairy skin.
- **References**

Experimental Section

Materials Characterization. Fourier-transform infrared spectroscopy (FTIR, Thermo Scientific, Nicolet iS10) was employed to characterize the molecular structure of protein-based silk films. The spectroscopic analysis encompassed the wavenumber range from 400 to 4000 cm^{-1}, employing a resolution of 4 cm^{-1} and each spectrum integrated over 16 scans. Transmittance measurements of the silk films were conducted using a UV-VIS spectrometer (UV-3150, Shimadzu, Japan) spanning the wavelength range from 400 nm to 2000 nm, using a sample with a thickness of 100 µm. X-ray diffraction (XRD) analysis was executed within the 2θ range of 5° to 54°, employing a scanning rate of 3° per minute with Cu Kα radiation (Bruker D8 Advance instrument, Germany). The parameters were set to an X-ray beam wavelength of 1.5418 Å, a voltage of 40 kV, and a current of 40 mA. The specimens were cut into 1 cm^2 squares, each with a uniform thickness of 100 µm, and mounted on the zero-background stand for analysis. Raman spectroscopy was carried out within the spectral range of 350 to 3500 cm^{-1} employing a backscattered confocal micro-Raman microscopy (LabRAM HR, Horiba, Japan). The spectrometer was configured to a groove density of 600 g/mm. The laser power and integration time for each spectrum are as follows: for the 532 nm spectra, the acquired power is 40 mW, and the integration time is 10 seconds; for the 633 nm spectra, the acquired power is 40 mW and the integration time is 120 seconds; for the 785 nm spectra, the acquired power is 100 mW and the integration time is 300 seconds.

Mechanical Measurements. Tensile tests were conducted on the fabricated silk films with varying $CaCl_2$/silk weight ratios formulation with a Universal testing meachine (UTM) (Zwick Z100, Germany). For each composition, 5 samples were tested (30 samples were investigated). The tests were conducted at a speed of 20 mm/min. The specimens were fabricated in an L-shape configuration, featuring a width of 20 mm, a thickness of approximately 200 µm, and an effective length of 40 mm. The Young's modulus was determined based on the data slope in (Δfore/Δstrain) the initial 0.5% strain region. To assess the resistance changes in the silk electrodes during elongation, a 20% strain was applied to the samples using a precision brushed motor (Thorlabs, MTS50A-Z8, USA) with an operating speed of 6 mm/min. Resistance measurements were obtained utilizing a precision benchtop multimeter (GDM-9061, GW Instek, Taiwan).

Sample Characterization. The surface morphology of the silk electronics was examined using a scanning electron microscope (Zeiss Gemini Supra 35 VP, LEO, Germany) operating at an acceleration voltage of 3 kV. To enable the imaging of samples, their surfaces were sputter-coated with a layer of gold-platinum approximately 10 nm thick. In tapping mode, surface topography was assessed using an atomic force microscope (Nanomagnetics Instruments, hpAFM, UK). The sessile drop method was employed to characterize the silk films' surface-wetting behavior. A liquid droplet (4 pL) was deposited on the substrate and allowed to spread for 10 sec. The side-view profile of the droplet-substrate interface was captured and the contact angle was measured (Theta Lite optical tensiometer, ON, Canada). The increasing contact angle was regarded as the lower wettability of the substrate.

Biodegradability Experiment. Silk film samples were prepared in square dimensions of 4 cm^2, each with a uniform thickness of 100 µm. For the soil substrate, regular soil rich in organic matter was sieved through a #18 sieve and oven-dried at 60 °C for 4 hours. In each circular plastic container, a total of 25 g of the prepared soil was placed, with the bottom and top diameters of the container measuring 4 and 7 cm, respectively. Following the silk films' placement in the soil's center, the soil was gently moistened once with 4 mL of distilled water. The plastic containers were securely sealed with aluminum foil to maintain appropriate air circulation, allowing for small openings. After an incubation period of 2 days, the soil was sieved once more to assess the silk film degradation rate visually.

Water Vapor Transmission Rate (WVTR). The WVTR of the silk films was assessed following the ASTM E96-95 standard protocol. A glass vial with a 4 mm opening was filled with 3 mL of distilled water and positioned on an 80 °C hotplate. Subsequently, the vial's opening was sealed with a 100 µm-thick silk film. Then, at regular intervals of 30 minutes over a 3-hour duration, the weight of the vial was measured. The WVTR was then calculated using Equation 1.

$$WVTR = \frac{\Delta m}{t \times A} \quad (2)$$

where Δm is the weight change during t hours, and A is the opening area covered with a sample.

Measurement of Adhesion Strength Using 90° Peeling Test. A 45 wt% $CaCl_2$/silk solution was poured onto a 1 cm × 15 cm Polyamide film and left overnight to allow for the evaporation of formic acid from the solution. The following day, one end of the Polyamide film, with the silk film on it, was attached to a dry glass surface, and the

other end was affixed to the mechanical grip of a mechanical tester (Zwick Z100, Germany). The test was conducted at a speed of 50 mm/min and a relative humidity of 75%. A similar method was employed to measure the adhesion force of Polyamide Kapton tape. The adhesion strength was subsequently calculated by dividing the resultant force by the width of the samples.

Skin-electrode Impedance Analysis. The skin and electrode interface impedance was investigated using an LCR meter (GW Instek, LCR-6002, Taiwan). The skin was cleaned and dried prior to the experiment. The silk skin electrodes as the measuring electrode and commercial "wet" silver/silver chloride (Ag/AgCl) electrodes (Beybi, China) were placed as the reference electrode with 5 cm spacing on the forearm of the volunteer. The impedance changes were recorded between 10-1000 Hz.

Sample Preparation. The red-dyed silk films were prepared by incorporating two droplets of a water-based red pigment ink into 20 grams of the pre-formulated silk solution. The polydimethylsiloxane (PDMS) skin-replica was fabricated by mixing Sylgard 184 silicone elastomer (DOW, USA) with its curing agent in a 10:1 ratio. The resulting mixture was stirred for 15 minutes and then poured onto a 60-grit sandpaper. This was followed by a degassing process and a curing period of 2 hours at 75 °C. After solidification in the oven, the PDMS was carefully detached from the sandpaper and utilized as the skin-replica.

Flexible and Wireless Signal Aquisition Unit Design. The schematic and printed circuit board (PCB) design for the biopotential unit were designed using Altium Designer 22.2.1, and the flexible board was manufactured commercially. The soldering process involved the application of solder paste (OM-5002, Alpha) to the flexible board using a stencil. Subsequently, the electrical components were positioned on the board. The board was then heated at 220°C for 5 minutes, which melted the solder paste and secured the components to the board. The flexible signal acquisition unit was attached on skin using double-sided silicone/acrylate thermoplastic elastomer medical tape (2477P), purchased from 3M (USA).

Humidity Adjustments. Humidity adjustments were carried out in a custom-built humidity chamber with a volume of 55 cm^3. This chamber was equipped with an ultrasonic piezoelectric humidifier, which served as the source of moisture, and a humidity sensor (HDC1080, Texas Instruments) that was connected to an Arduino board. To ensure the influence of humidity, all samples that were characterized at a specific humidity level were placed in the humidity chamber 90 minutes prior to the experiment. All humidity adjustments were conducted at an ambient temperature of 22.8 °C.

Thermal Analysis. Thermal analysis of the skin region covered with Silk-BioE was characterized using a Fluke Ti9 thermal camera (0.20 °C thermal sensitivity).

Artificial Sweat. The impact of perspiration was studied by applying artificial sweat (DIN 53160-2:2010/BS). This artificial sweat was composed of distilled water (98-99%), sodium chloride (≤1.0%), urea (≤0.5%), and lactate (≤0.5%).

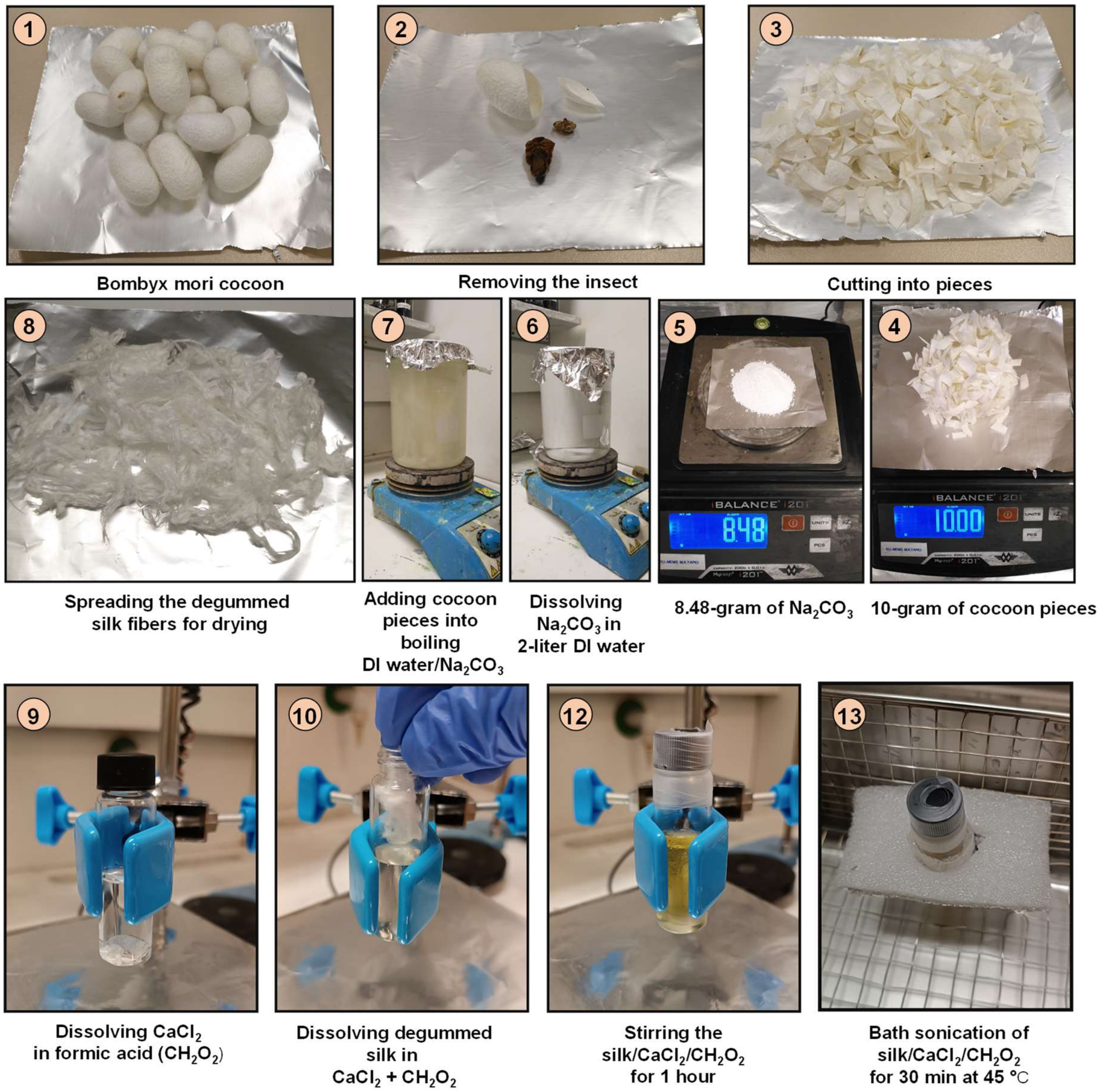

Figure S1. Process of degumming silk cocoons and silk solution preparation. The degumming process involves the following steps: 1) Procuring Bombyx mori cocoons. 2) Cutting the cocoons and removing the deceased insect. 3) Cutting the cocoons into approximately 1 cm^2 pieces using stainless-steel scissors. 4) Weighing out 10 g of the cut cocoon pieces. 5) Preparing a 0.04 M Sodium carbonate (Na_2CO_3) solution, which requires 8.48 g of Na_2CO_3. 6) Dissolving Na_2CO_3 in 2 liters of DI water and bringing it to a boil. 7) Boiling the cocoon pieces in the DI water/Na_2CO_3 solution for 45 minutes. 8) Thoroughly rinsing the degummed silk fibers with fresh DI water and allowing them to dry overnight in a fume hood. The silk solution preparation involves: 9) Dissolving calcium chloride ($CaCl_2$) in formic acid (HCOOH) using a magnetic stir bar. 10) Adding the degummed silk fibers to the $CaCl_2$/HCOOH solution. 11) Stirring the silk/$CaCl_2$/HCOOH solution for 1 hour using a magnetic stir bar. 12) Bath sonication of the silk/$CaCl_2$/HCOOH solution for 30 minutes at 45°C.

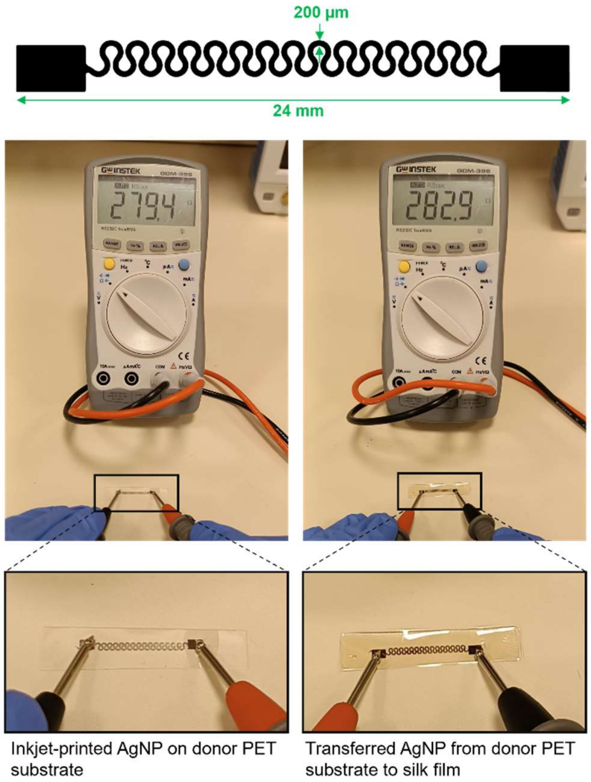

Figure S2. Impact of transfer printing on the integrity of printed AgNP patterns. The experiment aimed to evaluate the fidelity of printed silver nanoparticles (AgNP) structures before and after transfer printing. The resistance of a serpentine resistive design was measured as 279.4 Ω on the donor PET substrate before transfer and 282.9 Ω on the silk substrate after transfer. The slight variation of ~ 1.25% in resistance suggests minimal damage to the printed AgNP patterns during the transfer process, indicating good structural integrity.

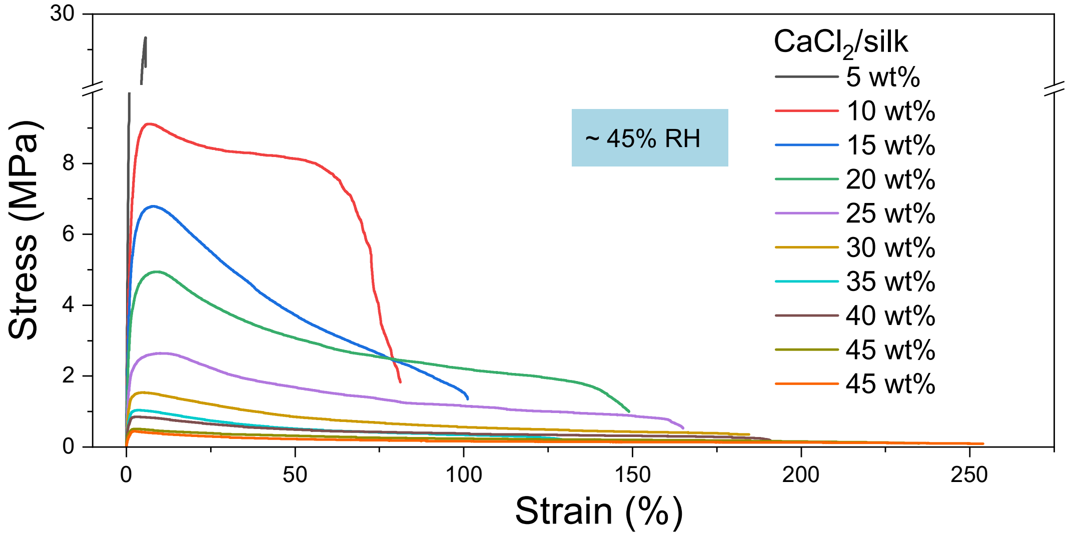

Figure S3. **Stress-strain curves for silk films modified with varying concentrations of $CaCl_2$, ranging from 5 wt% to 50 wt%.** The tests were conducted under approximately 45% relative humidity conditions. The curves demonstrate an increase in stretchability as the concentration of $CaCl_2$ increases.

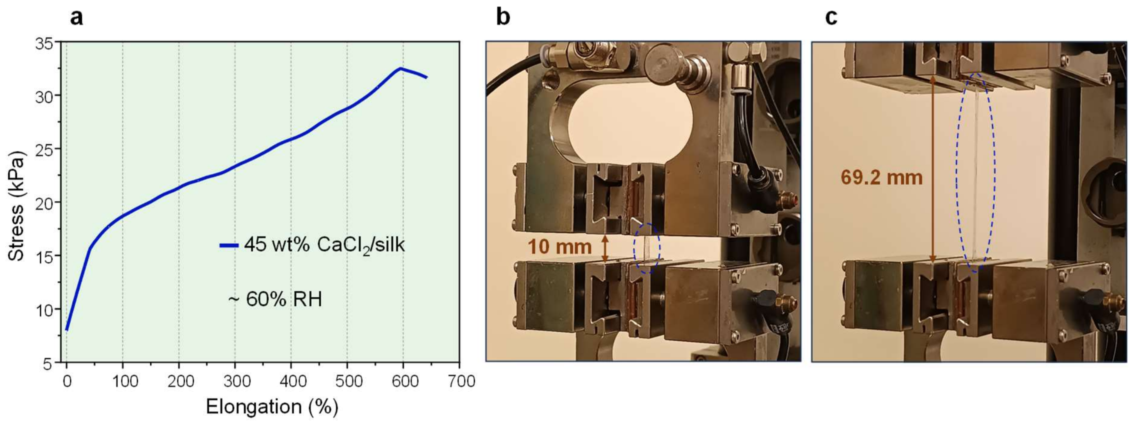

Figure S4. Stress-strain test of 45 wt% $CaCl_2$/silk films under 60% RH humidity. a) The resulting tensile stress-strain graph of the silk demonstrated an elongation exceeding 600% under a 60% RH humidity condition. b) The 45 wt% $CaCl_2$/silk film was attached to a mechanical testing machine with an initial length of 10 mm, and c) it stretched to a length of 69.2 mm, indicating an elongation of 592%.

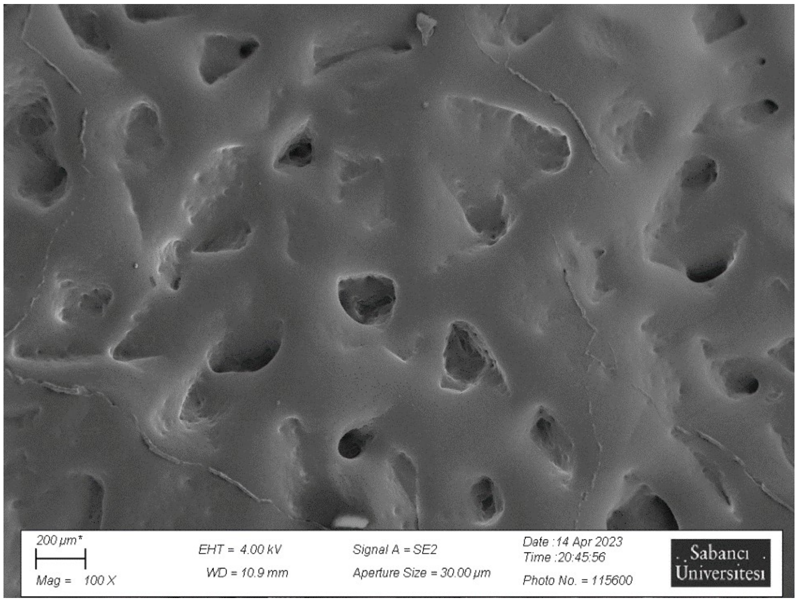

Figure S5. Surface topography of PDMS skin-replica. The SEM image represents the surface topology of the prepared PDMS skin-replica, highlighting pore dimensions of approximately 180 µm.

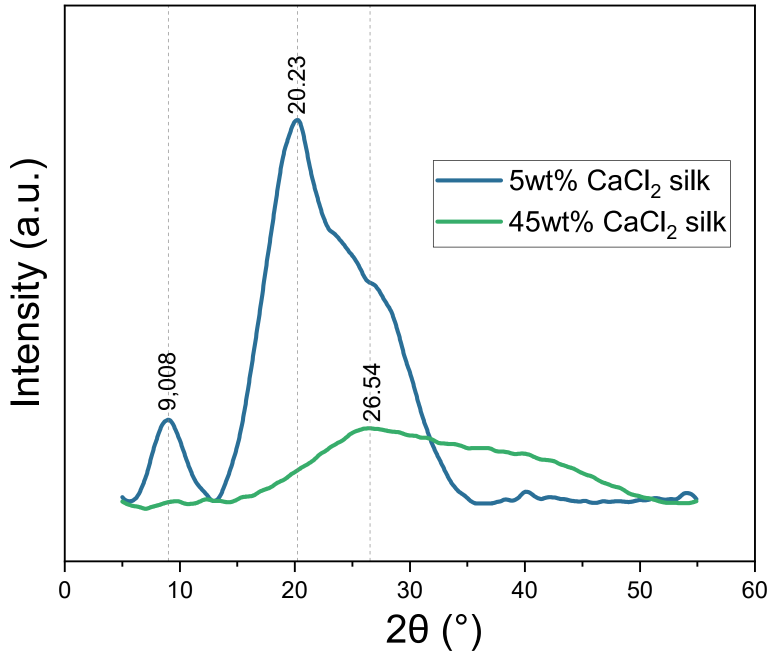

Figure S6. The X-ray diffraction (XRD) analysis of 5wt% and 45 wt% $CaCl_2$/silk films. The results indicate the transition from a crystalline to an amorphous structure in silk films as the $CaCl_2$/silk concentration increases from 5 wt% to 45 wt%. The silk film with a 45 wt% $CaCl_2$ concentration exhibits a broad peak at a 2θ scattering angle of 26.54°. In contrast, silk film with a 5 wt% $CaCl_2$ concentration displays two distinct peaks at 2θ scattering angles of 9.008° and 20.23°.

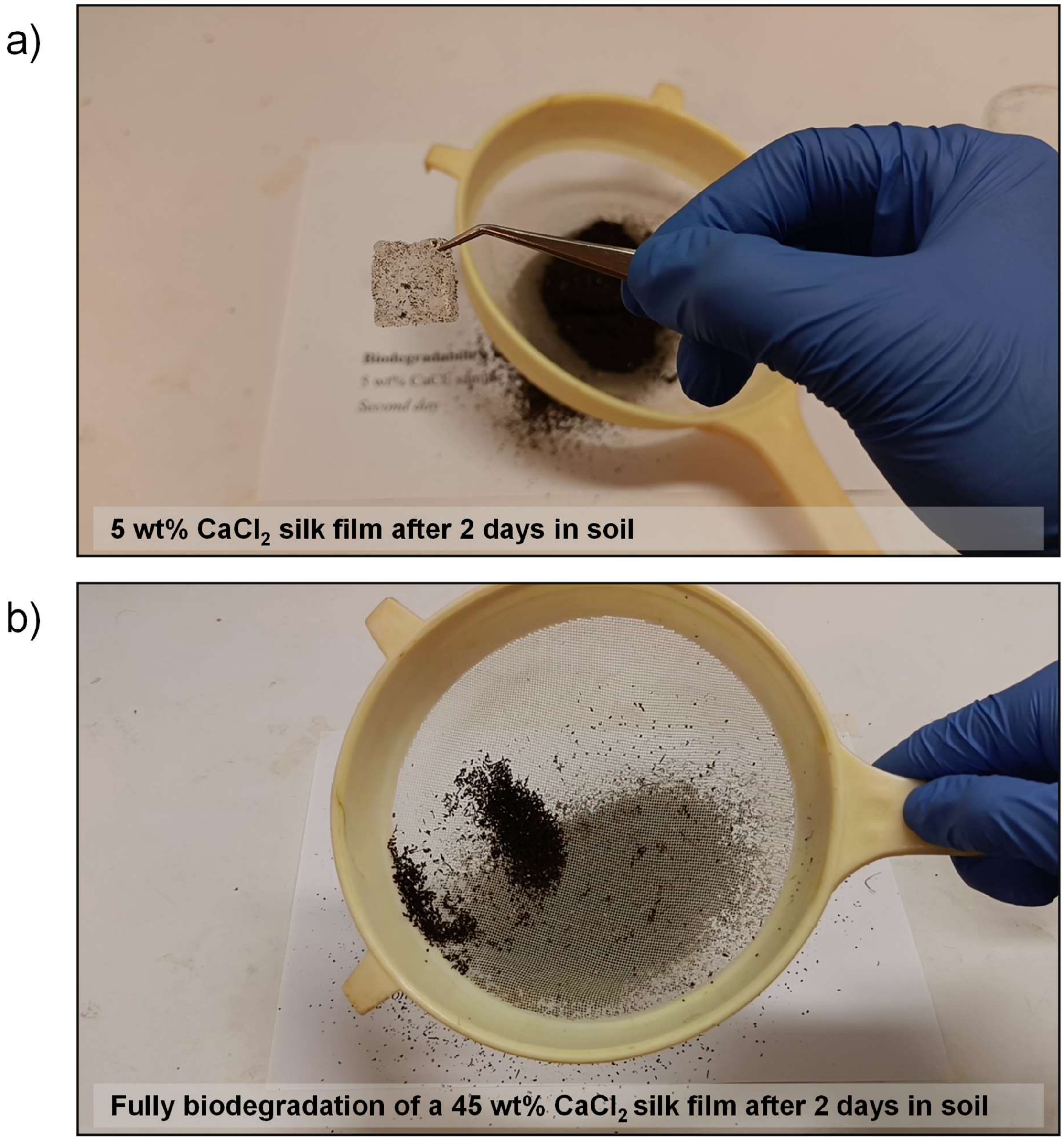

Figure S7. Biodegradability analysis of silk films in soil. Two silk films, one with a 5 wt% $CaCl_2$/silk ratio (n = 3) and the other with a 45 wt% $CaCl_2$/silk ratio (n = 3), both with a thickness of 100 µm, were buried in soil. After a period of 2 days, the soil was sifted to assess the condition of the silk films. The silk film with a 5 wt% $CaCl_2$ ratio remained intact (a), while the silk film with a 45 wt% $CaCl_2$ ratio was found to be completely degraded (b).

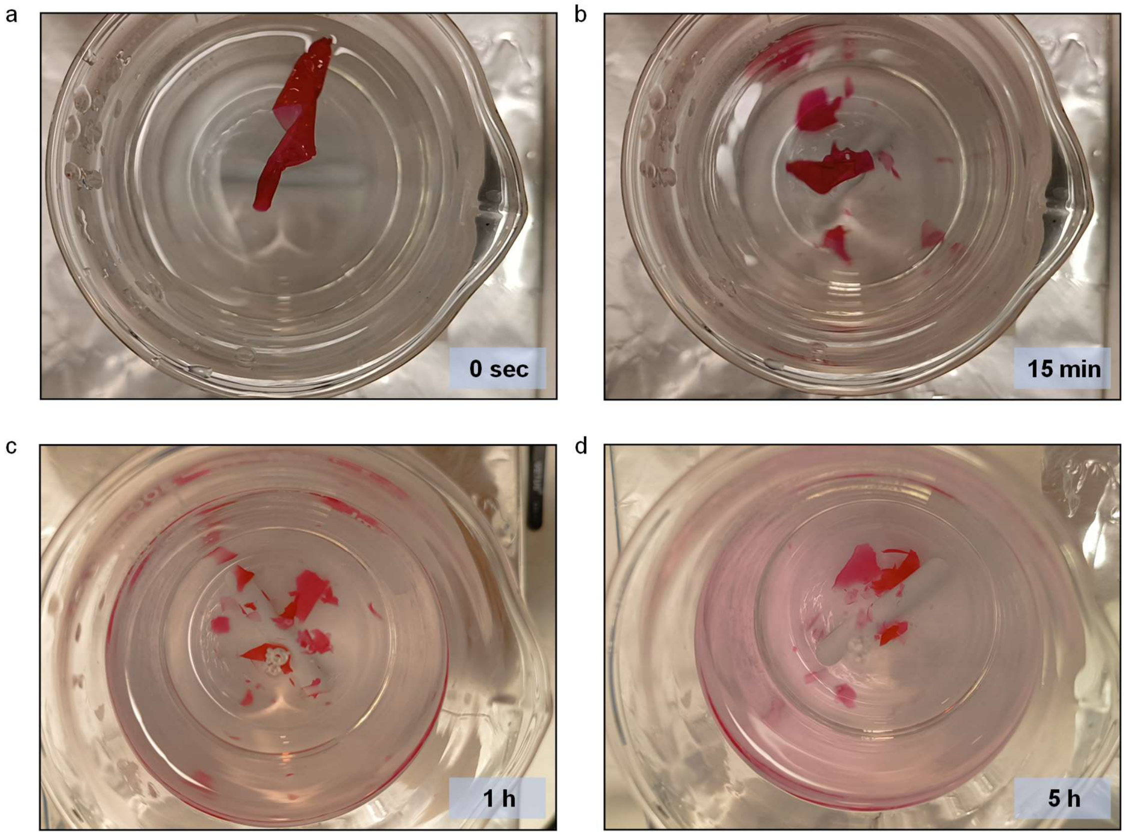

Figure S8. Water solubility analysis of silk films. A red-dyed silk film with a 45 wt% $CaCl_2$ ratio and a thickness of 100 μm was submerged in deionized water and subjected to continuous stirring for a duration of 5 hours. The figure shows the film a) immediately after being placed in the water, b) after 15 minutes, c) after 1 hour, and d) after 5 hours. The results highlight the water-insolubility of the 45 wt% $CaCl_2$ silk films, suggesting their potential suitability for applications in high humidity or sweat conditions.

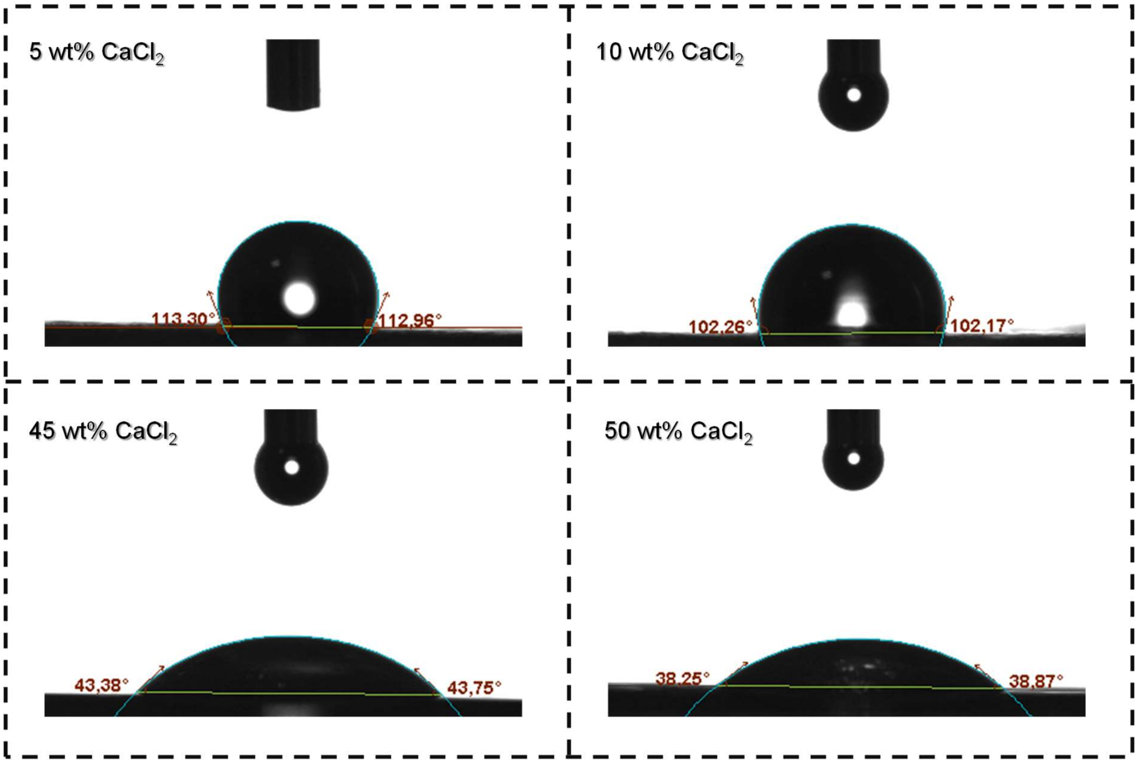

Figure S9. Surface wettability of silk films with varying $CaCl_2$/silk ratios, as determined by the sessile drop method using deionized water as the reference liquid. The findings suggest that as the $CaCl_2$/silk ratio increases, the contact angle of the silk films decreases. This suggests that silk films with higher $CaCl_2$ concentrations exhibit greater hydrophilicity compared to those with lower $CaCl_2$ concentrations.

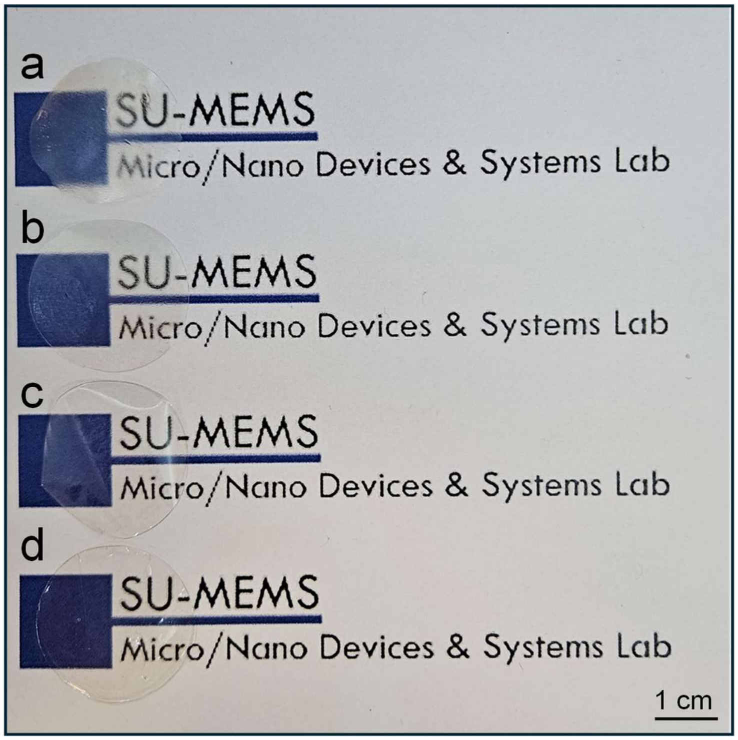

Figure S10. Optical transparency characterization of silk films with varying $CaCl_2$ concentrations. a) 5 wt%, b) 10 wt%, c) 25 wt%, and d) 45 wt% $CaCl_2$/silk. The images illustrate the decrease in transparency with increasing $CaCl_2$ concentration, consistent with UV-VIS spectroscopy results (film thickness, 100 μm).

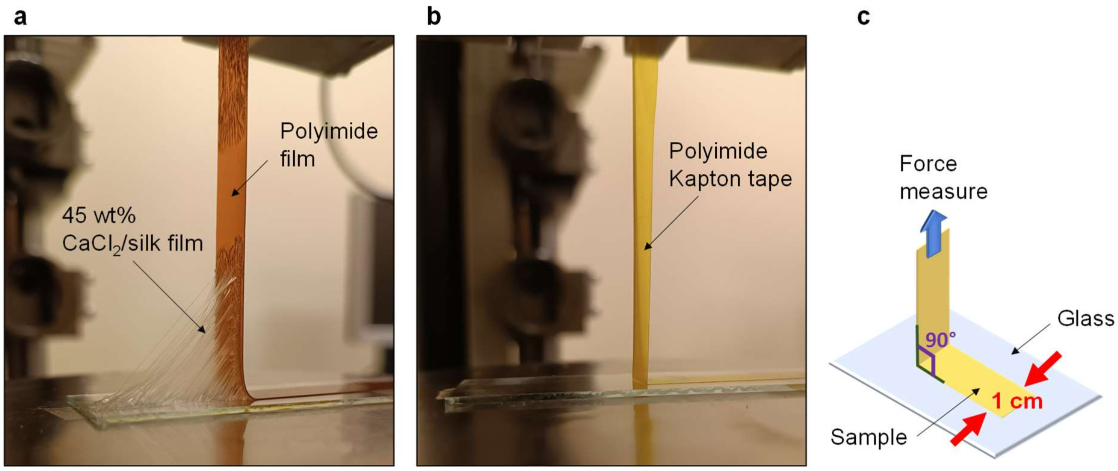

Figure S11. Adhesion strength measurement using 90° peeling test with respect to dry glass. a) Adhesion measurement of 45 wt% $CaCl_2$/silk film on Polyimide film. b) Adhesion measurement of Polyimide Kapton tape. c) Schematic representation of the 90° peel test setup.

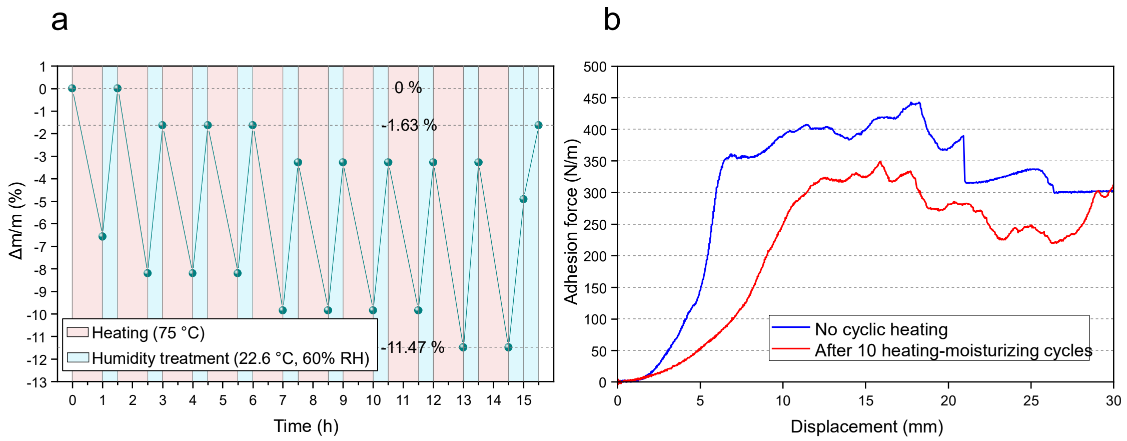

Figure S12. The influence of repeated heating and humidity on silk film adhesive properties. a) Weight variations of a 100 μm thick silk film were measured over 10 cycles of dehydration at 75 °C for one hour, followed by hydration at 60% relative humidity (RH). b) The adhesivity of a silk film subjected to 100 cycles of hydration and dehydration was compared to that of a silk film that did not undergo these cyclic conditions.

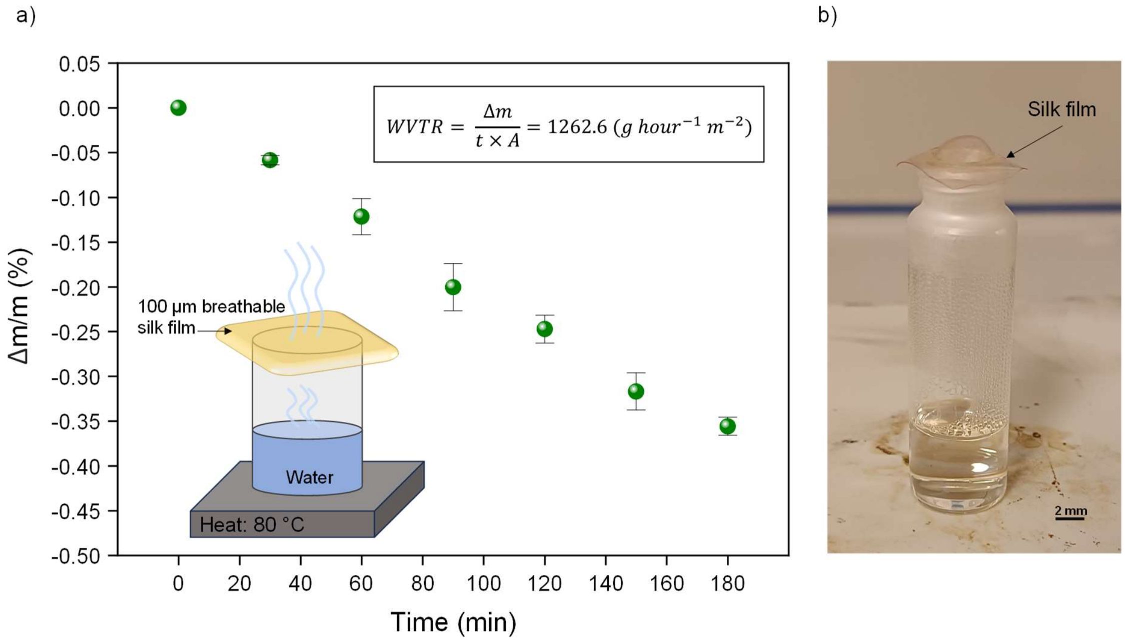

Figure S13. Water Vapor Transmission Rate (WVTR) of silk films. A glass vial with an opening diameter of 4 mm was sealed with 100 µm thick 45 wt% $CaCl_2$/silk film (*n* = 3) and then heated to 80 °C. a) Weight variations of the glass vial with the silk film at 30-minute intervals, demonstrating the loss of water from the vial that has evaporated through the silk film. The calculated WVTR was found to be 1262.6 g hour^{-1} m^{-2}. b) Image of the glass vial sealed with the silk film and placed on a hot plate set to 80 °C.

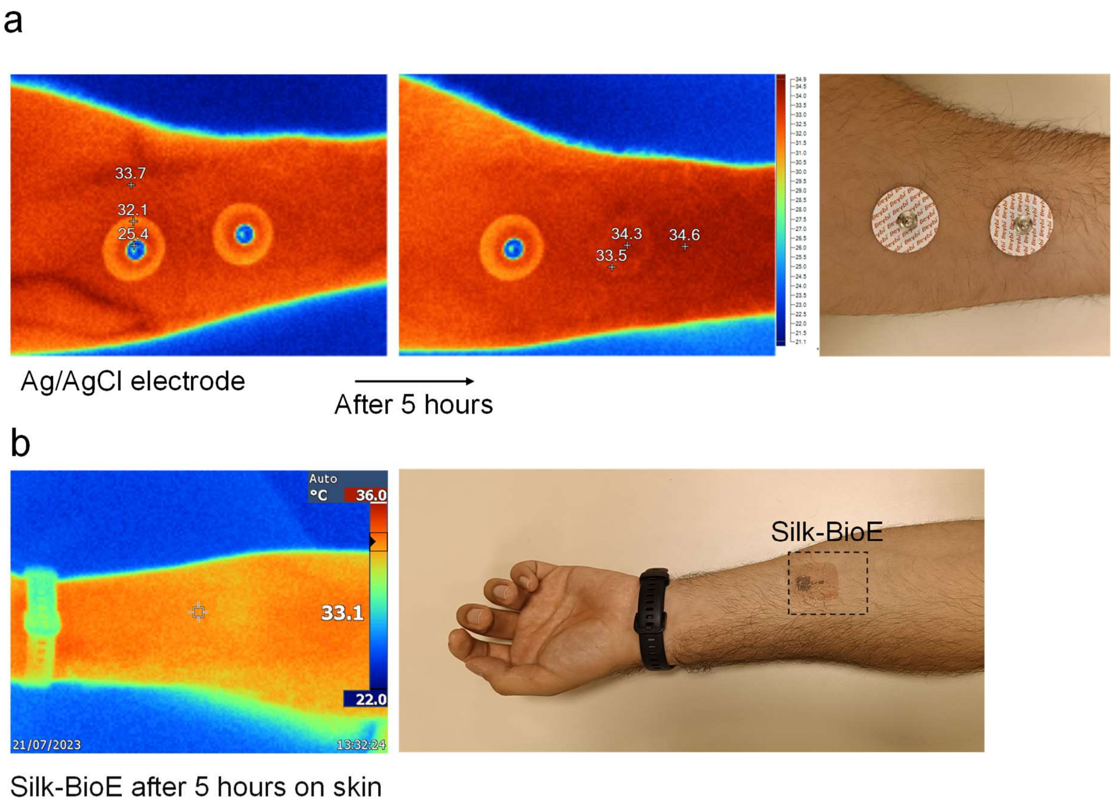

Figure S14. Thermal characterization of forearm skin following 5-hour application of commercial Ag/AgCl electrodes and Silk-BioE. Infrared imaging reveals no discernible temperature variation in the skin areas covered by either the commercial Ag/AgCl electrodes (a) or Silk-BioE (b). These findings highlight the breathability attribute of the developed silk bioelectronics, which are optimized for extended usage periods without inducing skin irritation, demonstrating a performance on par with a commercial electrode.

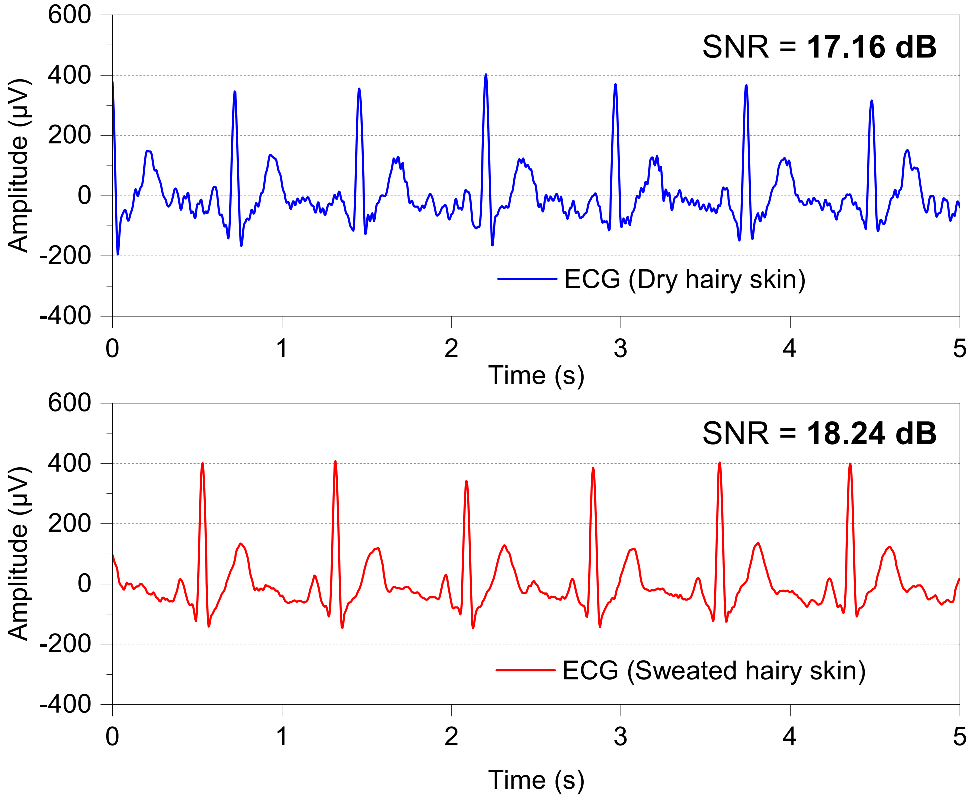

Figure S15. Influence of ionic composition of sweat on electrocardiography (ECG) recordings from hairy regions. Results demonstrate an increase in ECG signal signal-to-noise ratio (SNR) from 17.16 dB on dry hairy skin to 18.24 dB on hairy regions with artificial sweat. This improvement in signal quality can be linked to the abundance of sodium and chloride ions in sweat, which enhance the skin-electrode interface by facilitating electron transfer.

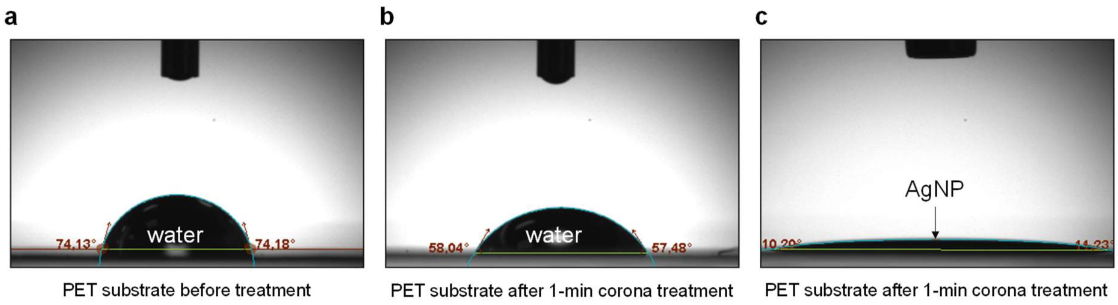

Figure S16. Impact of corona treatment on the surface wettability of polyethylene terephthalate (PET) substrates, optimized for inkjet-printing of silver nanoparticle (AgNP) ink. a) Contact angle between a non-treated PET substrate and DI-water, with an average value of approximately 74.15°. b) Contact angle between a PET substrate treated with corona for 1 minute and DI-water, which shows a decreased value of approximately 57.76°. c) Contact angle between a PET substrate treated with corona for 1 minute and AgNP ink, with an average value of 10.71°.

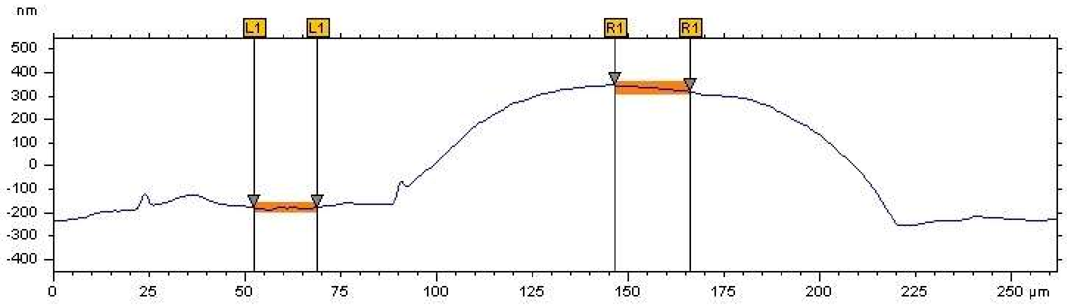

Figure S17. Profilometer result for a PET substrate with a single cycle of inkjet-printed AgNP. The measured thickness of the printed layer is approximately 514.45 nm.

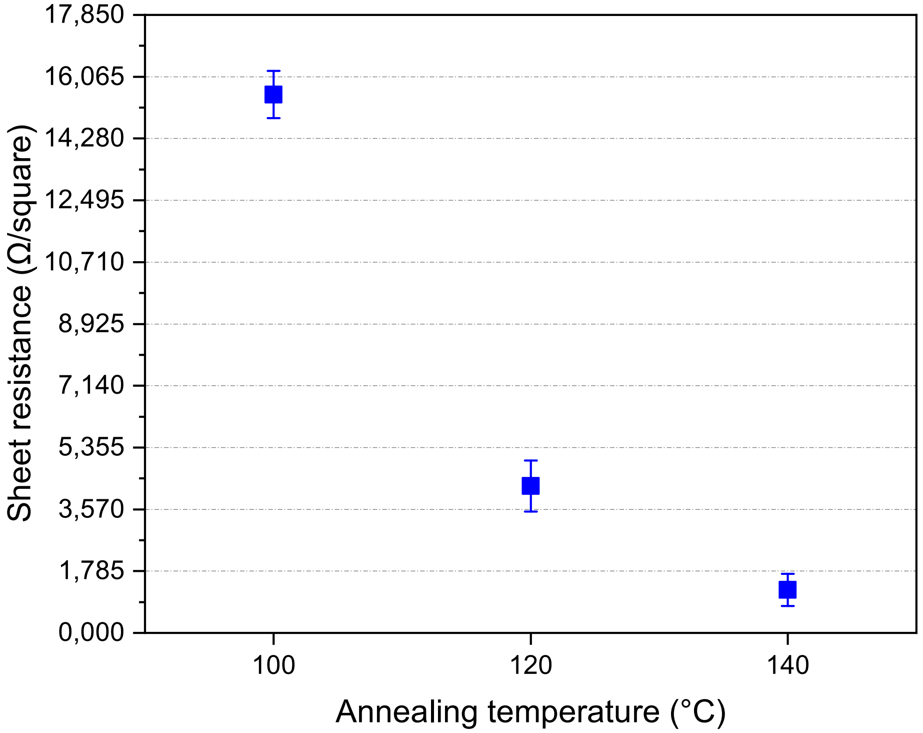

Figure S18. The impact of annealing temperature on the sheet resistance of inkjet-printed AgNP. The sheet resistances were measured as 15.55, 4.24, and 1.24 Ω/square at annealing temperatures of 100, 120, and 140 °C, respectively, for a duration of 20 minutes for all conditions.

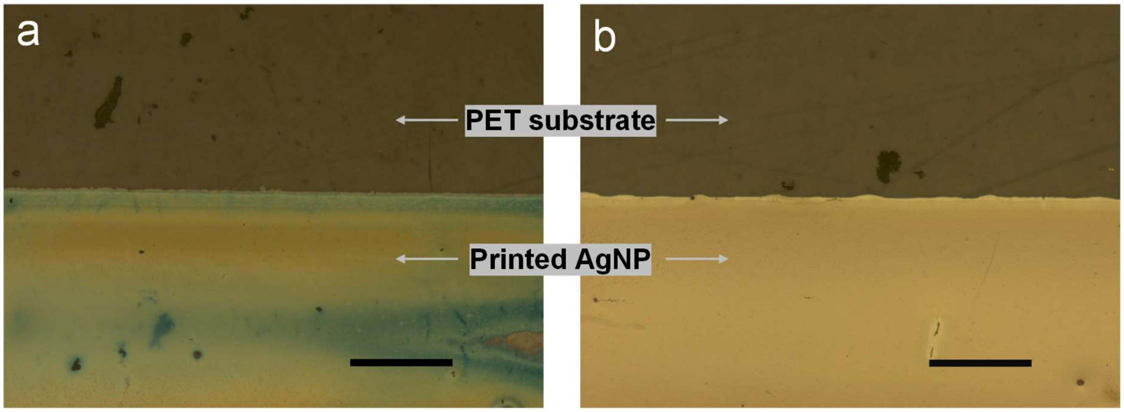

Figure S19. Impact of ambient air on the oxidation of inkjet-printed AgNP samples. a) Microscopic image of inkjet-printed AgNP ink on a PET substrate that was left in ambient air for 1 month. The sample underwent oxidation, and its resistance varied from 4.65 to 8.2 Ω. b) Microscopic image of inkjet-printed AgNP ink on a PET substrate that was preserved in a vacuum desiccator. The sample did not oxidize, and its resistance remained stable. These results suggest that samples patterned with AgNP have a long shelf-life under low humidity conditions. Scale bar, 400 μm.

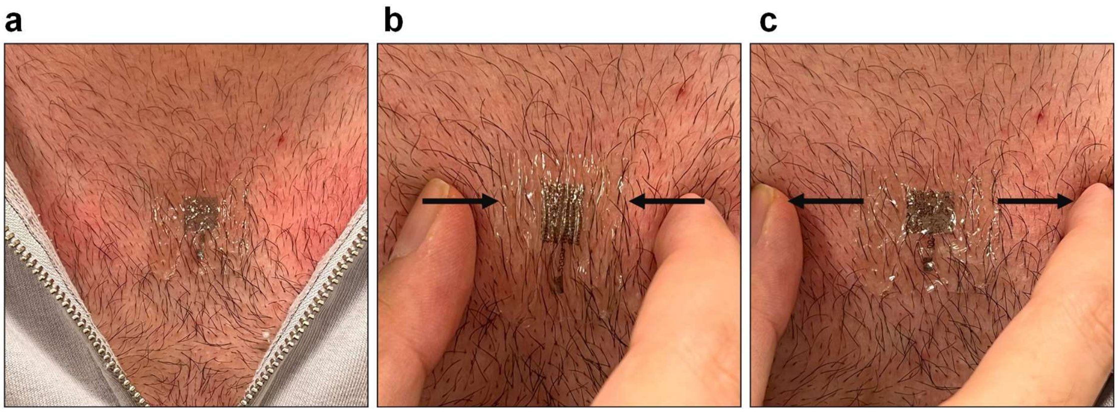

Figure S20. Feasibility of attaching the Silk-BioE to hairy skin areas due to its superior self-adhesiveness. The images show the Silk-BioE attached to the (a) chest, (b) under compression, and (c) under stretching.

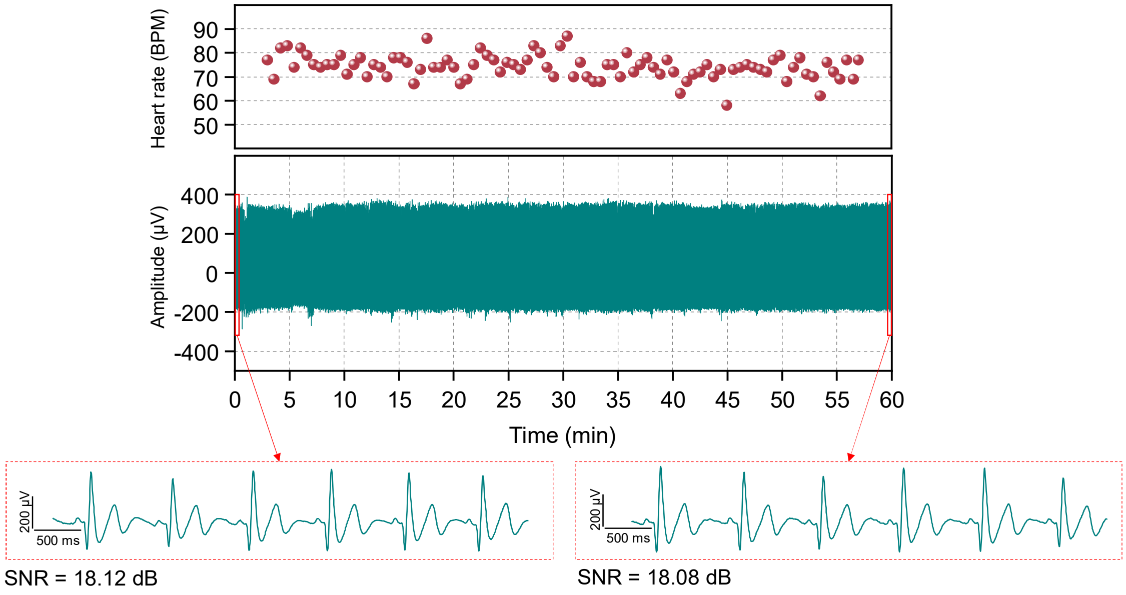

Figure S21. Performance assessment of Silk-BioE on hairy skin during prolonged perspiration. To simulate continuous sweating during intense physical activity, artificial sweat was applied to the Silk-BioE electrodes every 10 minutes over 1-hour ECG recording. The results revealed that SNR values at the beginning (18.12 dB) and end (18.08 dB) of the recording showed minimal variation. These findings indicate that Silk-BioE is suitable for prolonged use on hairy and perspiring areas of the body without significant degradation in signal quality.

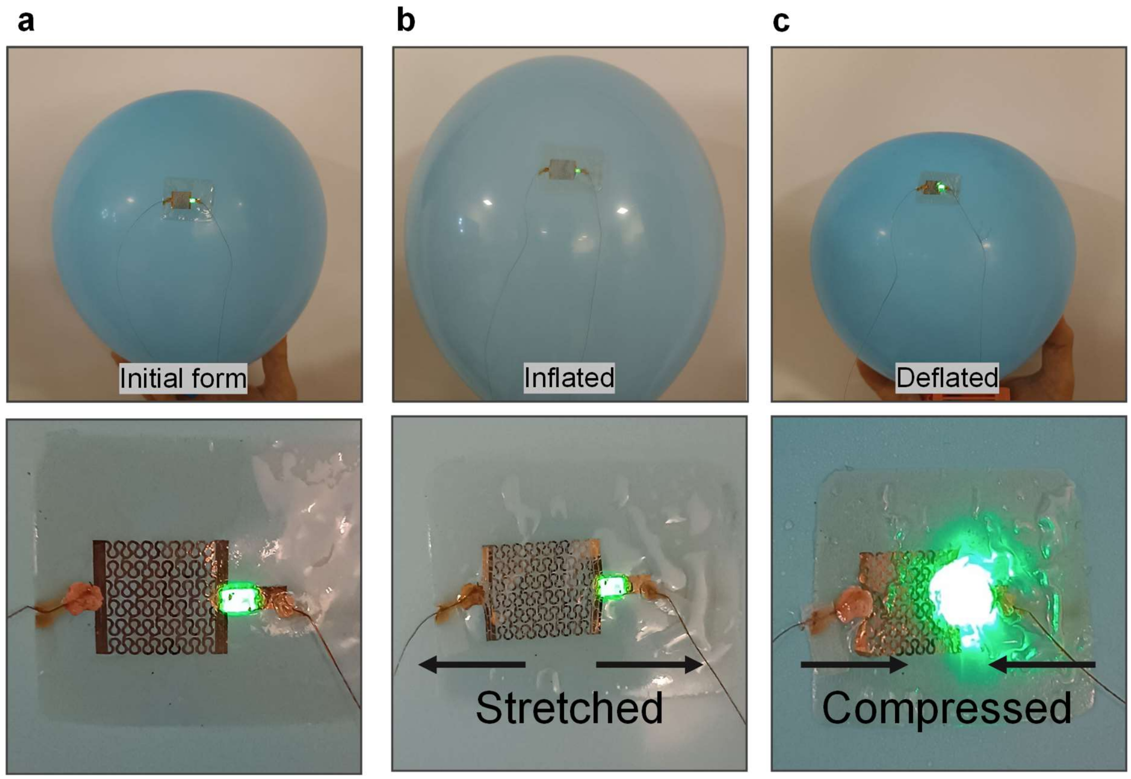

Figure S22. Electrical performance of Silk-BioE under conditions of stretching and compression attached on a balloon. The images depict an LED-integrated Silk-BioE attached to a balloon. (a) Silk-BioE after attachment to the balloon. (b) Silk-BioE stretched over the inflated balloon. (c) Silk-BioE in a compressed state after the balloon has been deflated, relatively to its initial form. The results demonstrate that the Silk-BioE maintains its electrical conductivity regardless of whether the balloon is inflated or deflated. The deformations experienced by the balloon can be likened to natural skin deformations, which involve stretching and compression.

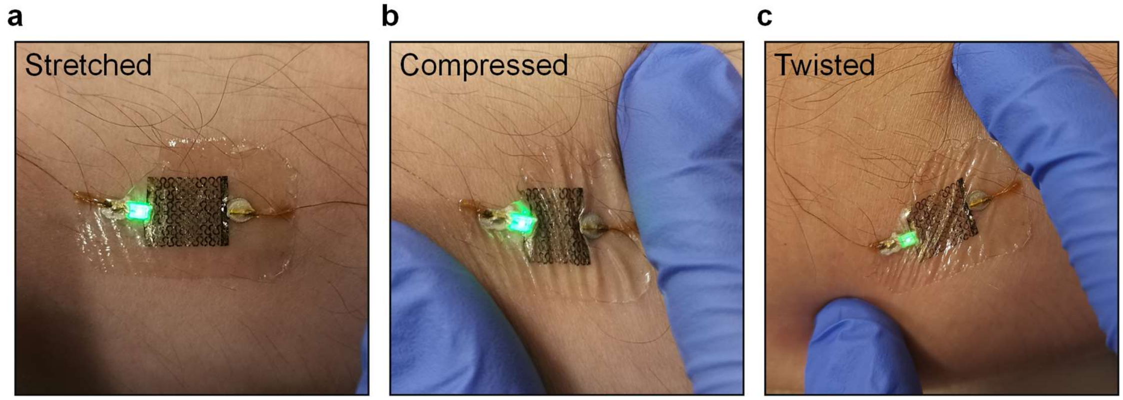

Figure S23. Electrical performance of an LED-integrated Silk-BioE applied to skin under various skin deformations. (a) stretching, (b) compression, and (c) twisting. The findings reveal that the Silk-BioE maintains stable electrical conductivity, even when subjected to natural skin deformations.

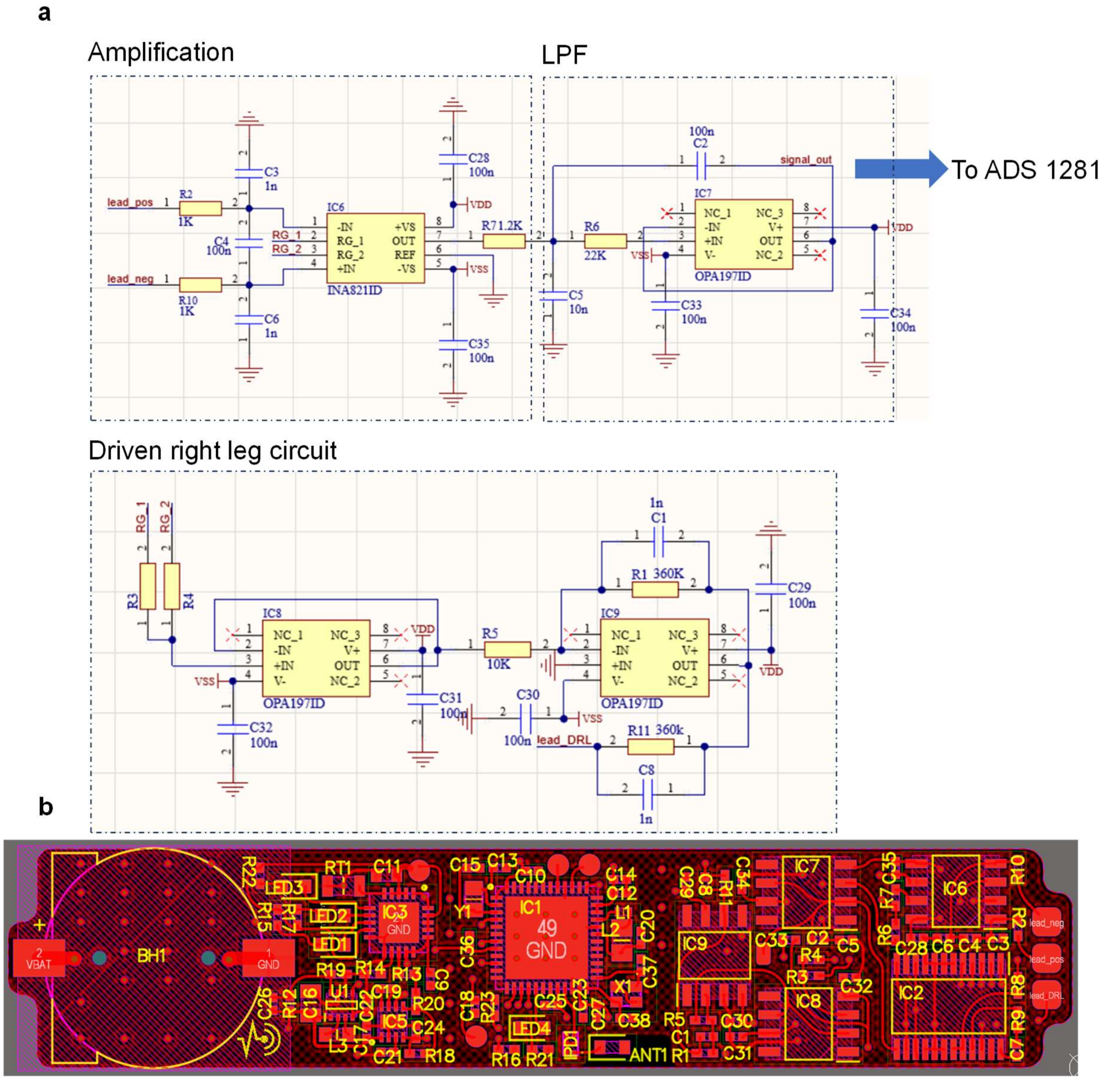

Figure S24. Design of the analog front end and PCB for the flexible, wireless biopotential signal acquisition unit. (a) Details of the analog front end of the unit, which includes an amplification stage, a low-pass filter (LPF), and a driven right leg (DRL) circuit. (b) The design of the printed circuit board (PCB), with the top layer interconnects colored in red and the bottom layer interconnects in blue.

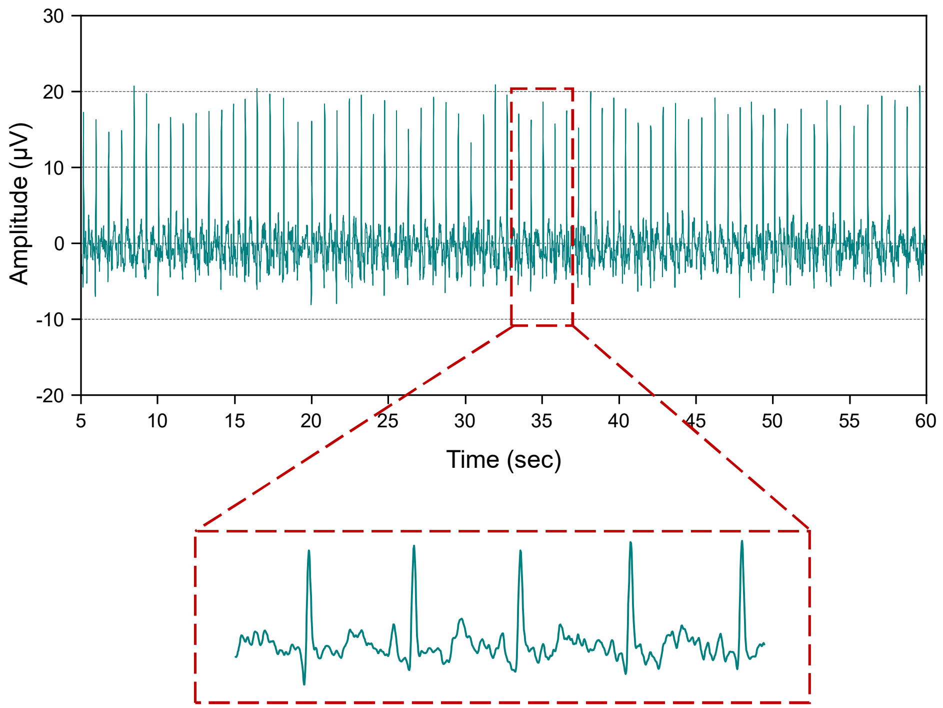

Figure S25. Single-arm ECG signal acquisition using Silk-BioE and flexible biopotential unit. Three Silk-BioE were aligned horizontally on the left arm of a participant. The ECG signal collected exhibited a peak amplitude of approximately 20 µV.

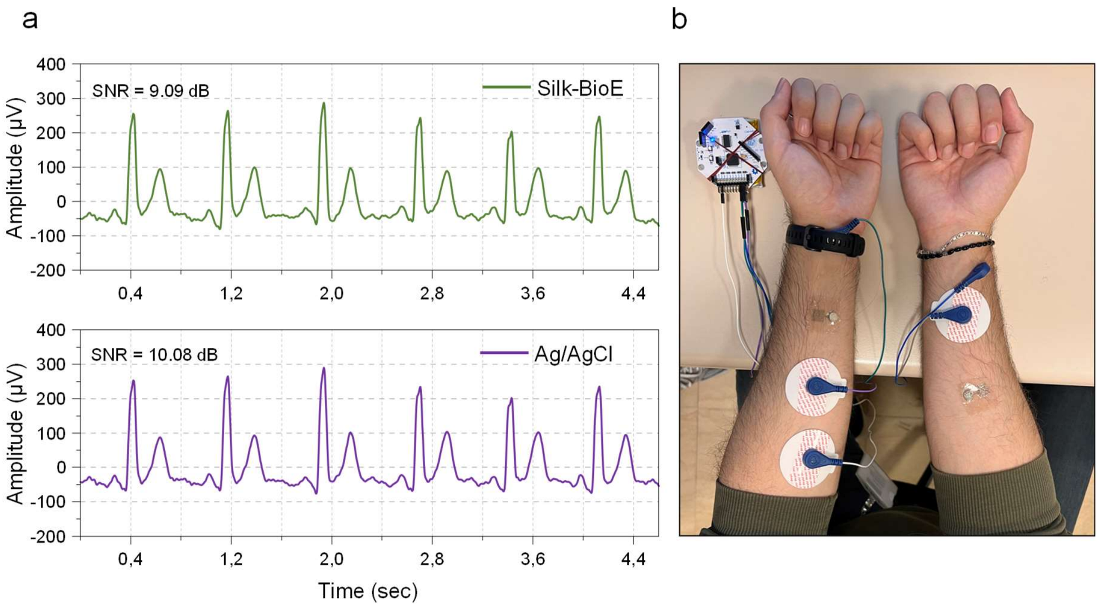

Figure S26. Simultaneous ECG recording using Silk-BioE and Ag/AgCl electrodes. a) The recorded ECG signals exhibit SNR values of 9.09 dB for Silk-BioE and 10.08 dB for Ag/AgCl, respectively. These signals demonstrate a Pearson's correlation coefficient of 99.4%. b) The figure illustrates the setup for ECG collection in a lead-I configuration, along with a commercial signal acquisition board (Cyton board, OpenBCI).

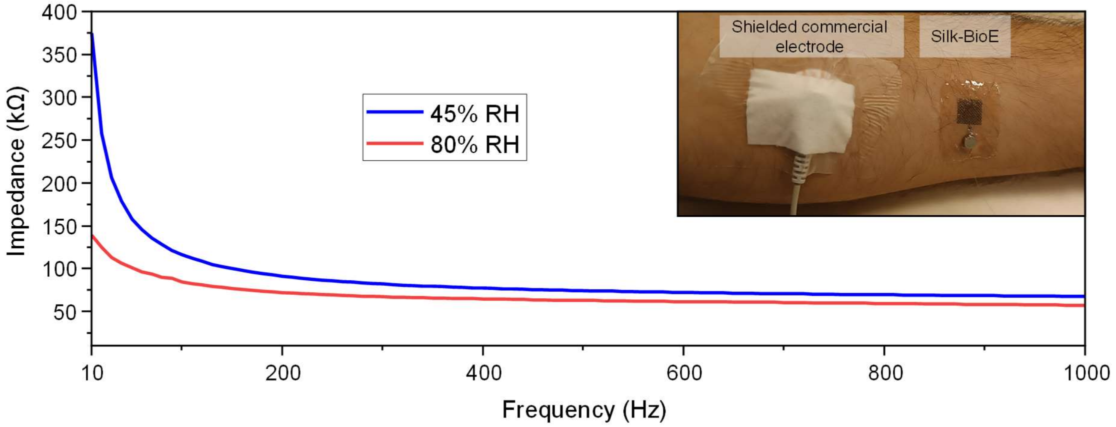

Figure S27. Effect of ambient humidity on the skin-electrode impedance of Silk-BioE at 45% and 80% RH. The effect of ambient humidity on the skin-electrode impedance of Silk-BioE was investigated at two relative humidity levels: 45% and 80% RH. Silk-BioE was positioned 5 cm away from a commercial Ag/AgCl reference electrode, which was covered with a waterproof medical-grade bandage to minimize humidity effects on the reference electrode. The experiment was conducted in a humidity chamber. The results indicate that increasing ambient humidity directly affects the elasticity of Silk-BioE, leading to an enhancement of the skin-electrode interface. Consequently, the skin-electrode impedance decreased from 375.28 kΩ at 45% RH to 139.16 kΩ at 80% RH (at 10 Hz).

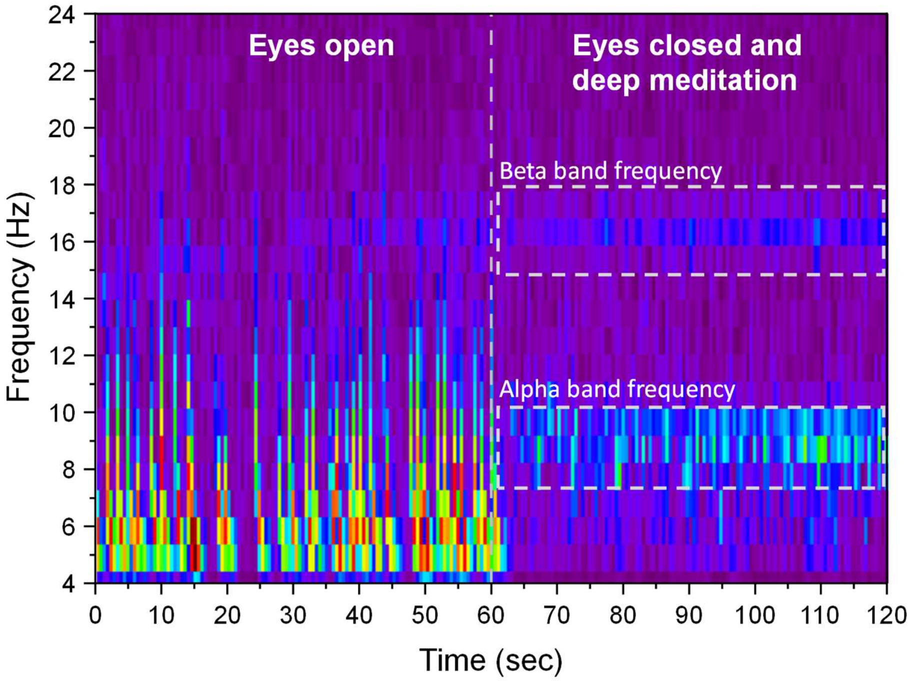

Figure S28. Fast Fourier Transform spectroscopy of EEG signals recorded using Silk-BioE, aimed at investigating beta-band frequencies during deep meditation phases. The participant was instructed to keep their eyes open and gaze forward for 60 seconds, followed by closing their eyes and performing basic mathematics for another 60 seconds. The results indicate that this sequence induced the generation of alpha bands due to eye closure, and beta bands due to the mental activity involved in performing mathematical tasks.

a b

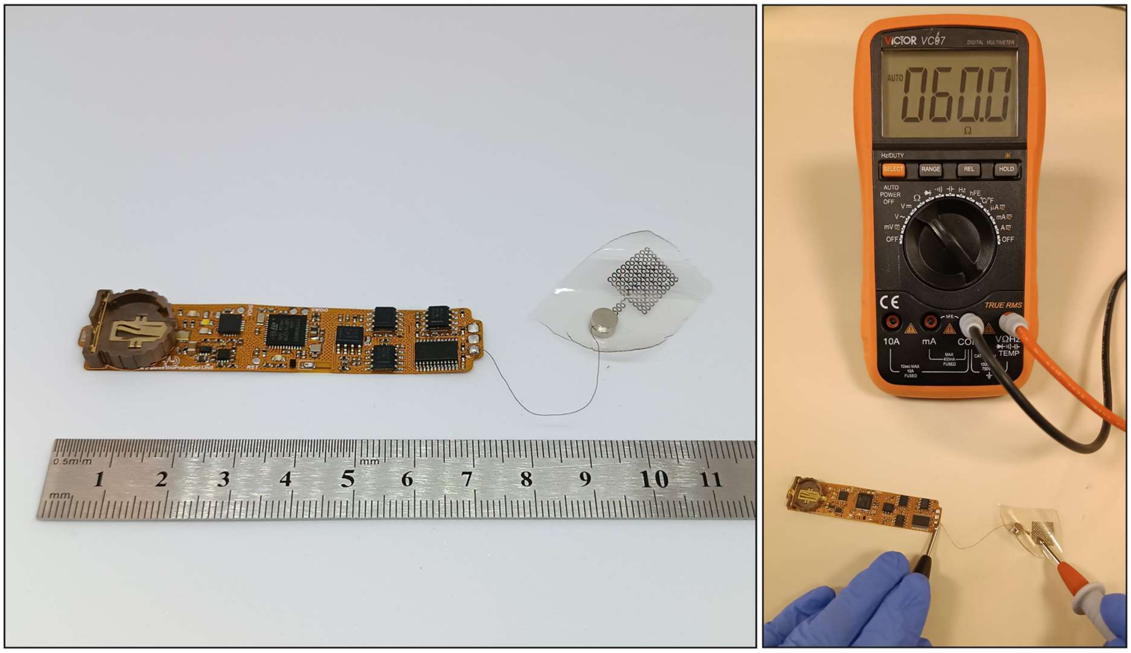

Figure S29. Connection between Silk-BioE and flexible signal acquisition unit. a) The Silk-BioE and signal acquisition unit were interfaced using a thin (30 µm) copper wire soldered to the flexible board. The opposite end of the wire was positioned on the Silk-BioE silver pad, with two conductive magnets (1.5 mm thick and 5 mm diameter) applying pressure to the wire against the pad from both the bottom and top. b) The electrical conductivity between the conductive AgNP serpentine mesh of the Silk-BioE and the flexible printed circuit board (FPCB) pad demonstrated a resistance of 60 Ω.

Table S1. Raman shift location and assignment of Silk Protein

Raman Shift [cm^{-1}]	Assignment [1, 2]
642 (1)	Tyrosine
827 (2)	Tyrosine
852 (3)	Tyrosine
887 (shoulder)	Proline
976 (small)	Sericin
943 (4)	α-Helix
1003 (small)	Phenylalanine
1025 (small)	Phenylalanine
1083 (6)	Sericin
1098 (shoulder)	Skeletal C_{α} –C_{β} stretching
1171 (7)	Tyrosine
1208 (shoulder)	Tyrosine, Phenylalanine
1230	Amide III
1335 (8)	Alanine
1399 (9)	Alanine
1414 (shoulder)	Glycine
1452 (10)	CH_3 asymmetric and CH_2 bending
1546 (11)	Tryptophan, Isoquinoline
1613 (12)	Tyrosine
1665	Amide I

Table S2. Qualitative comparison between Silk-BioE and previously reported various <u>non-silk-based</u> skin-like wearable bioelectronics for human physiological sensing.

Ref.	substrate	Conductive Material	Fabrication	Adhesive *[adhesivity source]*	Breathable	Transparent	Biodegradble	ECG	EEG	EMG	EOG
[1]	PDMS	Ag/AgCl/PEDOT:PSS	Stencil-printing	☑ *[PDMS-PEIE]*	✗	✗	✗	✓	✓	✓	✗
[2]	TPU	Ag/In/Ga/SIS	Contact-printing	☑ *[Acrylic adhesive]*	✗	☑	✗	✓	✓	✓	✓
[3]	Silicone elastomer	Cr/Au	Microfabrication	☑ [Self-adhesive]	✗	✗	✗	✓	✗	✗	✗
[4]	PET	CNT/PDMS/PEIE	✗	☑ *[PDMS-PEIE]*	✗	✗	✗	✓	✗	✗	✗
[5]	Ecoflex	Cr/Au	Microfabrication	☑ *[Adhesive silicone]*	☑	☑	✗	✓	✓	✓	✗
[6]	Silicone elastomer/PI	AgNP	Aerosol jet printing/ Microfabrication	☑ *[Adhesive elastomer]*	✗	✗	✗	✗	✗	✗	✓
[7]	TPU	AgNW	Dip-coating/ Laser cutting	☑ *[Liquid bandge]*	✗	☑	✗	✓	✗	✓	✗
[8]	PDMS	Galinstan (R)	Microfabrication	☑ *[Micropore commercial tape]*	✗	✗	✗	✓	✗	✗	✗
[9]	Silicone-based polymer	Carbon nanofiber/ Carbon black	Blend	☑ *[Self-adhesive]*	✗	✗	✗	✓	✓	✓	✗
[10]	PU nanomesh	Graphene	Electrospinning/ Laser scribıng	✗	☑	✗	✗	✓	✓	✗	✓
[11]	TPU/WPU	Mxene-CNT	Electrospinning/ Spraying	☑ *[Self-adhesive]*	☑	✗	✗	✓	✓	✓	✗
[12]	Ecoflex	Silve microparticle	Blend	☑ *[Micropillar structure]*	✗	✗	✗	✓	✓	✗	✗
[13]	WPU	PEDOT:PSS	Blend	☑ *[Self-adhesive]*	✗	✗	✗	✓	✓	✓	✗
[14]	PEDOT:PSS	Graphene	Spın-coat/ Etching	✗	✗	☑	✗	✓	✓	✓	✓
[15]	PI/PDMS	AgNW	Laser cutting/ Spray printing	☑ *[PDMS]*	☑	☑	✗	✓	✗	✗	✗
This work	Silk	AgNP	Inkjet-printing	☑ *[Self-adhesive]*	☑	☑	☑	✓	✓	✓	✓

Table S3. Inventory of functional components utilized in the construction of the flexible, wireless biopotential signal acquisition unit.

Component	Description	Value/Part number	Package
ANT1	Passive antenna	2450AT14A0100T	N/A
BH1	Battery holder	N/A	N/A
C1, C3, C6, C8	SMD capacitor	1n	402
C2, C4, C10, C12, C18, C23, C25, C28, C29, C30, C31, C32, C33, C34, C35, C36	SMD capacitor	100n	402
C5	SMD capacitor	10n	402
C7, C19	SMD capacitor	1u	402
C9, C14, C17, C20, C21	SMD capacitor	4.7u	402
C11	SMD capacitor	10u	603
C13, C15	SMD capacitor	15p	402
C16	SMD capacitor	47uF	805
C22	SMD capacitor	22uF	402
C24	SMD capacitor	2.2u	402
C26	SMD capacitor	4.3p	402
C27	SMD capacitor	100p	402
C37, C38	SMD capacitor	20pF	402
IC1	Bluetooth low energy (BLE)	STM32WB55CGU6	UFQFPN-48
IC2	Analog-to-Digital Converter	ADS1281IPW	TSSOP-24
IC3	Battery charger	MCP73871-1AAI_ML	QFN-20
IC5	Voltage inverter	LM27761DSGR	WSON-8
U1	Buck-Boost converter	TPS631000DRLR	VSON-10
IC6	Instrumentation Amplifier	INA821ID	SOIC-8
IC7, IC8, IC9	Op-amp	OPA197ID	SOIC-8
IPD1	Low Pass Filter	MLPF-WB55-01E3	N/A
L1	SMD inductor	10nH	402
L2	SMD inductor	10uH	805
L3	SMD inductor	2.2uH	603
LED1	SMD LED	White	603
LED2	SMD LED	RED	603
LED3	SMD LED	Green	603
LED4	SMD LED	Blue	603
R1, R11	SMD resistor	360K	402
R2, R10, R15, R17, R21, R22, R23	SMD resistor	1K	402
R3, R4	SMD resistor	2.75K	402
R5, R8, R9, R13, R16	SMD resistor	10K	402
R6	SMD resistor	22K	402
R7	SMD resistor	1.2K	402
R12	SMD resistor	510K	402
R14	SMD resistor	100K	402
R18	SMD resistor	36K	402
R19	SMD resistor	91K	402
R20	SMD resistor	56K	402
RT1	SMD resistor	NTCS0805E3103FLT	805
X1	Crystal Oscillator 32 MHz	NX2016SA-32M-EXS00A-CS06465	N/A
Y1	Crystal Oscillator 32.768 kHz	NX2012SA-32.768K-STD-MUB-1	N/A

Supplementary Videos

Movie S1. Biodegradability of silk substrates in soil. In this movie, the natural degradation of silk substrates in soil environment by tailoring the wt% of $CaCl_2$ is demonstrated. While the 5 wt% $CaCl_2$ silk substrates remain intact, the 45 wt% $CaCl_2$ silk substrates completely decompose in 2 days.

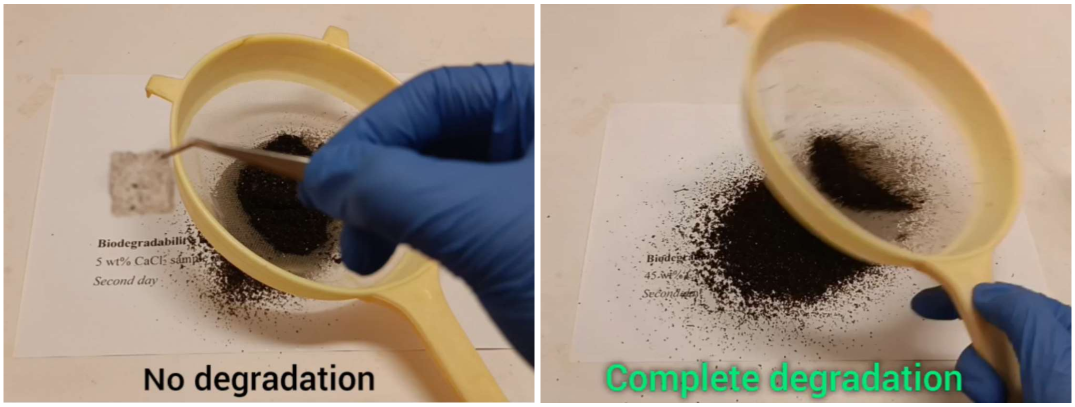

Movie S2. Excellent self-adhesiveness and robust performance of Silk-BioE in recording electrocardiography signals (ECG) on hairy regions of the body and under severe perspiration. This film demonstrates the exceptional performance of Silk-BioE in recording ECG signals under the combined effects of body hair and perspiration.

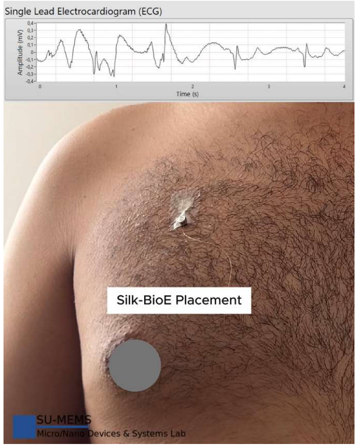

Movie S3. Silk-BioE on skin under running water. The movie showcases the water-insoluble properties of Silk-BioE on skin as well as its superior self-adhesiveness, as it withstands direct running water multiple times without delamination.

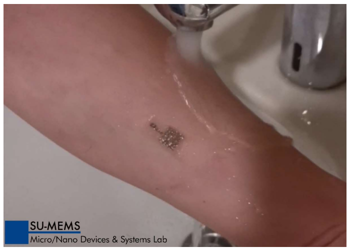

Movie S4. Removal of Silk-BioE from the skin through a simple washing process. This movie showcases the effective removal of the highly adhesive Silk-BioE from the skin. The process involves gently rubbing the skin under water, which robustly washes away the Silk-BioE.

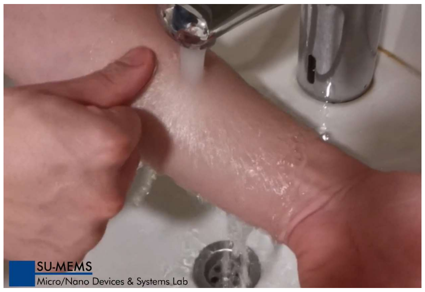

Movie S5. Silk-BioE on skin, undergoing various phases of compression, stretching, and twisting. This movie illustrates how Silk-BioE accurately mimics skin deformations such as compression, stretching, and twisting, while maintaining its original shape.

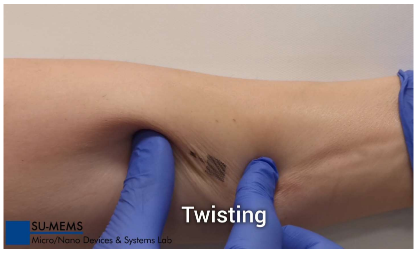

Movie S6. Evaluation of removal comfort: Silk-BioE vs. commercial Ag/AgCl electrodes on hairy skin. This video illustrates the effortless removal of Silk-BioE from hairy areas of the body, causing no discomfort to the wearer. In contrast, the commercial electrode, with its synthetic adhesive layer, results in significant pain during removal from similar regions.

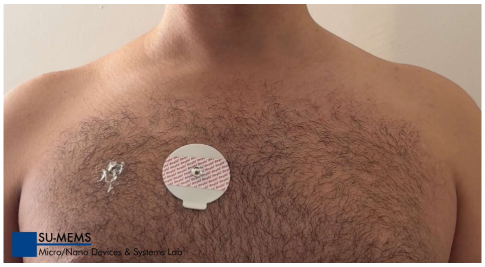

References

[1] K. Wang, U. Parekh, T. Pailla, H. Garudadri, V. Gilja, T. N. Ng, *Adv Healthc Mater* **2017**, *6*, DOI 10.1002/adhm.201700552.

[2] M. Reis Carneiro, C. Majidi, M. Tavakoli, *Adv Funct Mater* **2022**, *32*, DOI 10.1002/adfm.202205956.

[3] Y. Kim, M. Mahmood, Y. Lee, N. K. Kim, S. Kwon, R. Herbert, D. Kim, H. C. Cho, W. Yeo, *Advanced Science* **2019**, *6*, DOI 10.1002/advs.201900939.

[4] Y. Yamamoto, D. Yamamoto, M. Takada, H. Naito, T. Arie, S. Akita, K. Takei, *Adv Healthc Mater* **2017**, *6*, DOI 10.1002/adhm.201700495.

[5] L. Tian, B. Zimmerman, A. Akhtar, K. J. Yu, M. Moore, J. Wu, R. J. Larsen, J. W. Lee, J. Li, Y. Liu, B. Metzger, S. Qu, X. Guo, K. E. Mathewson, J. A. Fan, J. Cornman, M. Fatina, Z. Xie, Y. Ma, J. Zhang, Y. Zhang, F. Dolcos, M. Fabiani, G. Gratton, T. Bretl, L. J. Hargrove, P. V. Braun, Y. Huang, J. A. Rogers, *Nat Biomed Eng* **2019**, *3*, 194.

[6] S. Mishra, Y.-S. Kim, J. Intarasirisawat, Y.-T. Kwon, Y. Lee, M. Mahmood, H.-R. Lim, R. Herbert, K. J. Yu, C. S. Ang, W.-H. Yeo, *Sci Adv* **2020**, *6*, DOI 10.1126/sciadv.aay1729.

[7] W. Zhou, S. Yao, H. Wang, Q. Du, Y. Ma, Y. Zhu, *ACS Nano* **2020**, *14*, 5798.

[8] Y. Li, Y. Luo, S. Nayak, Z. Liu, O. Chichvarina, E. Zamburg, X. Zhang, Y. Liu, C. H. Heng, A. V. Thean, *Adv Electron Mater* **2019**, *5*, DOI 10.1002/aelm.201800463.

[9] Q. Tian, H. Zhao, X. Wang, Y. Jiang, M. Zhu, H. Yelemulati, R. Xie, Q. Li, R. Su, Z. Cao, N. Jiang, J. Huang, G. Li, S. Chen, X. Chen, Z. Liu, *Advanced Materials* **2023**, *35*, DOI 10.1002/adma.202211236.

[10] Y. Qiao, X. Li, J. Wang, S. Ji, T. Hirtz, H. Tian, J. Jian, T. Cui, Y. Dong, X. Xu, F. Wang, H. Wang, J. Zhou, Y. Yang, T. Someya, T. Ren, *Small* **2022**, *18*, DOI 10.1002/smll.202104810.

[11] Y. Hao, Q. Yan, H. Liu, X. He, P. Zhang, X. Qin, R. Wang, J. Sun, L. Wang, Y. Cheng, *Adv Funct Mater* **2023**, *33*, DOI 10.1002/adfm.202303881.

[12] F. Stauffer, M. Thielen, C. Sauter, S. Chardonnens, S. Bachmann, K. Tybrandt, C. Peters, C. Hierold, J. Vörös, *Adv Healthc Mater* **2018**, *7*, DOI 10.1002/adhm.201700994.

[13] L. Zhang, K. S. Kumar, H. He, C. J. Cai, X. He, H. Gao, S. Yue, C. Li, R. C.-S. Seet, H. Ren, J. Ouyang, *Nat Commun* **2020**, *11*, 4683.

[14] Y. Zhao, S. Zhang, T. Yu, Y. Zhang, G. Ye, H. Cui, C. He, W. Jiang, Y. Zhai, C. Lu, X. Gu, N. Liu, *Nat Commun* **2021**, *12*, 4880.

[15] Y. Ling, G. Zhao, Y. Su, Q. Wu, Y. Xu, Z. Chen, B. Arends, O. Emeje, G. Huang, J. Xie, Z. Yan, *Adv Funct Mater* **2023**, *33*, DOI 10.1002/adfm.202302681.
